# Real-Life Comparative Analysis of Robotic-Assisted Versus Laparoscopic Radical Prostatectomy in a Single Centre Experience

**DOI:** 10.3390/cancers16213604

**Published:** 2024-10-25

**Authors:** Stefano Salciccia, Valerio Santarelli, Giovanni Battista Di Pierro, Francesco Del Giudice, Giulio Bevilacqua, Giovanni Di Lascio, Alessandro Gentilucci, Roberta Corvino, Valentina Brunelli, Greta Basile, Carlo Maria Scornajenghi, Lorenzo Santodirocco, Luca Gobbi, Davide Rosati, Martina Moriconi, Valeria Panebianco, Fabio Massimo Magliocca, Daniele Santini, Mattia Alberto Di Civita, Flavio Forte, Marco Frisenda, Giorgio Franco, Alessandro Sciarra

**Affiliations:** 1 Department Materno Infantile e Scienze Urologiche, University Sapienza, 00161 Rome, Italy; stefano.salciccia@uniroma1.it (S.S.); va.santarelli@uniroma1.it (V.S.); giovannibattista.dipierro@uniroma1.it (G.B.D.P.); francesco.delgiudice@uniroma1.it (F.D.G.); giulio.bevilacqua@uniroma1.it (G.B.); giovanni.dilascio@uniroma1.it (G.D.L.); alegenti@yahoo.it (A.G.); roberta.corvino@uniroma1.it (R.C.); valentina.brunelli@uniroma1.it (V.B.); greta.basile@uniroma1.it (G.B.); carlomaria.scornajenghi@uniroma1.it (C.M.S.); lorenzo.santodirocco@uniroma1.it (L.S.); luca.gobbi@uniroma1.it (L.G.); davide.rosati@uniroma1.it (D.R.); martina.moriconi@uniroma1.it (M.M.); giorgio.franco@uniroma1.it (G.F.); 2 Department of Radiology, University Sapienza, 00161 Rome, Italy; valeria.panebianco@uniroma1.it (V.P.); fabiomassimo.magliocca@uniroma1.it (F.M.M.); 3 Department of Oncology, University Sapienza, 00161 Rome, Italy; daniele.santini@uniroma1.it (D.S.); mattiaalberto.dicivita@uniroma1.it (M.A.D.C.); 4 Urologic Division, Vannini Hospital, 00177 Rome, Italy; flavio.forte@figliesancamillo.it (F.F.); marco.frisenda@uniroma1.it (M.F.)

**Keywords:** prostatic neoplasm, radical prostatectomy, robotic surgery, laparoscopic surgery

## Abstract

**Background**: The advantage of a robotic-assisted (RARP) over a laparoscopic (LRP) approach in radical prostatectomy (RP) remains to be demonstrated. **Aim**: The aim of the study is to use a homogeneous population in real life and single primary surgeon surgery to analyze the oncological and functional results based on the type of surgical approach and pathological features. **Methods**: This is a prospective trial on non-metastatic prostate cancer (PCa) patients considered after a multidisciplinary decision to conduct a RP, using a RARP or LRP approach. A real-life setting was analyzed at our Urological Departments using homogeneous criteria for the management of PCa cases and a single surgeon experience on 444 cases (284 LRP and 160 RARP). **Results**: Mean operating time was significantly lower in RARP (153.21 ± 25.1 min) than in LRP (173.33 ± 44.3 min) (*p* < 0.001). In cases submitted to an extended lymph node dissection (eLND), the mean number of lymph nodes removed was 15.16 ± 7.83 and 19.83 ± 4.78, respectively, in LRP and RARP procedures (*p* < 0.001), but positive lymph nodes (pN1) were similarly found in 15.8% of LRP patients and 13.6% of RARP patients (*p* = 0.430). Surgical margins (SM) positivity was not significantly higher in the RARP group (20.0%) when compared to the LRP group (15.9%) (*p* = 0.145). During the postoperative follow-up, a biochemical recurrence (BCR) was detected in 14.4% and 7.5% of cases in the LRP and RARP group, respectively, (*p* = 0.014). Better results of PAD tests at 3-month intervals using the RARP approach (mean pad weight 75.57 ± 122 g and 14 ± 42 g, respectively, in LRP and RARP (*p* < 0.01)) were described. **Conclusions**: In the comparison between the RARP and LRP approach, a clear advantage of the robotic approach is a significant reduction in operating times, days of hospitalization, and postoperative catheterization compared to laparoscopic surgery. It is not possible to describe any certain oncological advantage both in terms of surgical margins and pathological lymph nodes removed. In RARP cases a reduction to the limit of significance is described in terms of biochemical recurrence. RARP produces a more rapid recovery of urinary continence at 3 months postoperatively without significant advantages in terms of erective potency recovery.

## Simple Summary

In the comparison between the RARP and LRP approach, a clear advantage of the robotic approach is a significant reduction in operating time, days of hospitalization, and duration of postoperative catheterization compared to laparoscopic surgery. It is not possible to describe any certain oncological advantages, both in terms of surgical margins and pathological lymph nodes removed. In RARP cases a reduction to the limit of significance is described in terms of biochemical recurrence. RARP produces a more rapid recovery of urinary continence at 3 months postoperatively without significant advantages in terms of erective potency recovery.

## 1. Introduction

Robotic-assisted radical prostatectomy (RARP) has become the most widely used robotic intervention and the most frequently performed technique for the treatment of non-metastatic prostate cancer [1,2,3,4,5,6].

However, its advantage over the laparoscopic (LRP) approach remains to be demonstrated, but its greater simplicity of execution and shorter learning curve make it favored [1,2,3,4]. The European Urological Association (EAU) recommends informing patients who are candidates for prostatectomy that no surgical approach (laparoscopic versus robotic) has clearly demonstrated superiority in terms of both oncological and functional results [7]. However, several clinical trials have shown an advantage of RARP over LRP in terms of recovery of sexual potency and surgical margins (SMs) status in organ-confined PCa [1,2,3,4,5,6,8,9,10,11,12,13,14,15,16]. The presence of SM+ represents the most immediate indication of failure for RP to achieve patient cure. However, more and more frequently its treatment is not immediate and is being replaced by vigilant monitoring, delaying treatment in case of early biochemical recurrence [8,9,10,11,12,13,14,15,16].

Even in this setting, a randomized study can allow us to compare two homogeneous populations to verify significant differences between the different approaches. However, the limit of the randomized study is the distance that can be produced from a real-life situation as well as the difficulty of execution. Moreover, a study that reaches a significant number of RP samples through the coexistence of different surgeons with different approaches and experiences introduces a relevant factor of variability that can reduce the validity of the results [1,2,3,4,5,6,17,18,19,20,21,22,23,24,25].

In the present study we report on the long-term case history of a population of patients with non-metastatic PCa considered suitable, according to EAU guidelines and after multidisciplinary discussion, for radical prostatectomy, performed by a single primary surgeon with experience of over 10 years. This population represents a homogeneously treated sample in a perfect real-life situation at a high-volume institution for the treatment of PCa and with a multidisciplinary (MDT) group regulating decisions relating to the management of each individual patient.

The aim of the study is to use this homogeneous population in real life to analyze the oncological and functional results based on the type of surgical approach but also based on the clinical and pathological characteristics of the neoplasms.

## 2. Materials and Methods

This is a prospective trial on non-metastatic PCa patients considered after MDT discussion to perform RP, either RARP or LRP. A real-life setting was analyzed at our Urological Departments and a single center experience is reported.

### 2.1. Population

Patients with a histologically confirmed diagnosis of non-metastatic PCa considered for RP as primary treatment and submitted to surgery by a single primary surgeon (AS), were consecutively enclosed in the analysis. The analysis was approved by our internal ethical committee and all patients gave their informed consent for each procedure. All diagnostic and therapeutic procedures reflected our routine clinical practice in a high-volume department for the management of PCa disease. All diagnostic and therapeutic decisions for the management of patients and selection for surgery were discussed in an MDT group. In particular for low-risk PCa patients, possible options between active surveillance, surgery, and radiotherapy were offered and presented with their possible advantages, limitations, and side effects. In patients at intermediate or high risk, the two options, surgery and radiotherapy, were presented equally to the patient. Inclusion criteria were that patients had no distant metastases at clinical staging, had a histological diagnosis of adenocarcinoma, an estimated life-expectancy of ≥10 years, and had RP as their chosen primary treatment option. Exclusion criteria were if patients had had pelvic radiation therapies, chemotherapies, androgen deprivation therapies, or treatment with any other medication that could influence prostate tumor growth. From January 2013 to December 2023, 444 consecutive PCa patients who underwent RP in our Department of Urology by a single surgeon (AS), corresponding to defined inclusion and exclusion criteria, were included in our analysis (Supplementary Table S1).

### 2.2. Clinical Parameters

The whole population of 444 cases is described in Supplementary Table S1. Patients, after prostatic specific antigen (PSA) increase, underwent a multiparametric magnetic resonance imaging of the prostate (mpMRI) with PI-RADS score determination. Targeted samples on sites indicated by mpMRI were associated with random samples in each prostate lobe. Using PSA determination and imaging (mpMRI, CT and bone scan, PET–CT scan), a clinical staging and risk category (D’Amico and EAU classification) assessment was homogeneously performed before surgery. Validated predictive nomograms in terms of risk for positive lymph node involvement were used [13,17]. In cases with familiarity and high-risk PCa, a genetic evaluation for pathogenic variants (PV) of Damage DNA Repair (DDR) genes was performed on surgical samples as part of a prospective trial.

### 2.3. Pathologic Evaluation

All histological specimens from prostatic biopsy and RP were analyzed by our uro-pathologist with lengthy experience in the PCa field. Gleason score and grade groups according to the World Health Organization (WHO)/ISUP 2014 guidelines at biopsy and at surgery, as well as pathological staging using the TNM classification and SM status were routinely described in all cases. SMs were considered positive when carcinoma was transected by an inked SM; either in a setting of organ-confined or extracapsular disease.

### 2.4. Surgical Procedure

Surgical technique was not assigned randomly, and no specific selection was performed regarding the two approaches. As routine clinical practice in our department, each procedure (RARP and LRP) was discussed with the patient and was performed by the same primary surgeon (AS) who had high expertise (>10 years for LRP and >5 years for RARP) in each approach, consistent with best practices. All surgical procedures (LRP and RARP) were performed using the same intraperitoneal standard technique for RP, starting from the bladder neck and proceeding to the prostate apex. A nerve-sparing (NS) (intrafascial, monolateral, or bilateral) procedure was performed after discussion with the patient based on mpMRI results, risk classes, the risk of extracapsular disease, and the probability of maintaining potency. In particular, for either RARP or LRP: (1) intrafascial NS was performed in cases with low- or intermediate-risk classes of PCa, low risk of ipsilateral extracapsular PCa extension, and good preoperative sexual potency; (2) the surgical intraperitoneal technique was equal using either RARP or LRP; (3) patients with a high clinical risk of ipsilateral extracapsular disease were excluded from a NS surgery; (4) extended lymph node dissection (eLND) was performed in the intermediate-risk class cases with a ≥5% probability for positive nodes based on predictive nomograms and in all high-risk cases; (5) intra-operative evaluation of SM was not performed. Intra and perioperative complications were reported, in particular anastomotic leakage, lymphocele, rectal injury, blood transfusion, and urethral stricture. Operative time, catheterization time, and postoperative hospitalization days were also reported.

### 2.5. Functional Analysis

Post-operative functional complications, such as urinary incontinence (UI), urethral stricture, and erectile dysfunction (ED) were analyzed during a 12-month follow-up. A persistent urinary leakage ≥5 g at a 24-h pad test defined an instance of UI. The use of a postoperative pelvic floor rehabilitation and pad test modifications during treatment was described, such as the need for an artificial sphincter procedure. A clinically significant ED was defined as an International Index of Erectile Function-5 (IIEF-5) score below 10. Postoperative tadalafil rehabilitation was performed in all cases submitted to NS procedure.

### 2.6. Oncological Outcomes

All patients were followed at regular intervals (every 3 months for the first 2 years and then every 6 months) to determine time to biochemical (blood-test confirmed total PSA ≥ 0.2 ng/mL), radiological (radiologically confirmed at PET CT scan and/or mpMRI), local, or distant recurrence, as recommended by current EAU guidelines. The decision for adjuvant therapy to surgery or early biochemical progression was taken homogeneously in all cases after discussion in the MDT and based on the EAU guidelines.

### 2.7. Statistical Analysis

For statistical analysis, the SPSS Statistics version 27.0 program was adopted. Descriptive statistics, such as number of cases, mean ± SD, median, and range were used. A Mann–Whitney test or ANOVA one-way test were used for the comparison of quantitative data and pairwise intergroup comparisons of variables. For comparison of qualitative data, Fisher’s exact test and a chi-square test were applied. Univariate and multivariate Cox proportional analysis for clinical and pathological parameters were used. Statistical significance was evaluated at *p* < 0.05. Kaplan–Meier analysis to define the cumulative rate of risk for biochemical progression was performed in the population stratified on the basis of different variables.

## 3. Results

Baseline characteristics of the whole population of 444 cases considered for RP are described in Supplementary Table S1. 284 patients (64.0%) were submitted to a laparoscopic approach (LRP) and 160 patients (36.0%) to a robotic-assisted surgery (RARP). The mean age of the population was 67.49 ± 6.53 with a range of 47–73 years, and the mean preoperative total PSA was 8.61 ± 5.74, ranged between 3.0 and 64.0 ng/mL.

An intermediate- and high-risk PCa was present in 45.0% and 23.0% of cases, respectively. An eLND was performed in 135 (30.4%) cases (mean number of removed lymph nodes: 17.22 ± 6.81), whereas an NS technique was used in 127 (28.6%) cases. At the final pathological evaluation an extracapsular disease (pT3) was found in 44.6% of cases (pT3a 33.8% and pT3b 10.8%). SM+ were found in 77 (17.3%) cases and positive lymph nodes (pN1) in 20 cases (14.8%). Mean surgical operative time was 160.05 min (range 90–300), mean time for hospitalization was 3.41 ± 0.56 days (range 3–7 days), and mean time of catheterization was 10.27 days (range 6–16 days). During the first post-operative 12-month follow-up, rates of urethral stricture, blood transfusion, and lymphocele were 2.7%, 0.5%, and 1.8%, respectively. Mean IIEF-5 changed from a preoperative 20.75 ± 5.83 to a postoperative 10.41 ± 24.10. A no-PADS status was obtained in 91.6% of cases after surgery.

Mean postoperative follow-up was 56.4 ± 37.8 months (range 12–120) with a 11.9% rate of biochemical recurrence (BCR) at a mean time of 18.67 ± 24.57 months. Adjuvant therapy was prescribed in 11.9% of cases, radiotherapy (RT) in 8.3%, and RT + androgen deprivation therapy (ADT) in 3.6%.

### 3.1. Differences in Pathological, Oncological and Functional Outcomes According to Surgical Approach

Clinical parameters such as preoperative PSA, prostate tumor size, and ISUP grading were not significantly (*p* > 0.50) different between cases submitted to RARP and LRP. A higher percentage (13.8%) of cases submitted to RARP were clinically staged T3 when compared to cases submitted to LRP (5.3%) (*p* < 0.001). Patients undergoing RARP were significantly younger (median 66 vs. 69 years; *p* < 0.001) and had a slightly but significantly higher Charlson Comorbidity Index (CCI) when compared to LRP patients (*p* < 0.001) (Table 1).

The distribution of PCa risk classes did not significantly (*p* = 0.849) differ between the two groups. In cases submitted to mpMRI, PIRADS 4 and 5 lesions were more frequent in the RARP group than in the LRP group (56.5% and 24.6% vs. 47.1% and 18.6%, respectively; *p* = 0.042) (Table 1). At biopsy, the percentage of positive cores did not significantly differ between the two groups (*p* = 0.772)**.** The percentage of bilateral positive biopsies was higher in the LRP group (*p* < 0.01) (Table 1).

The percentage of cases submitted to an NS procedure was 21.5% and 41.3%, respectively, (*p* < 0.01), whereas the percentage of cases submitted to eLND was 26.8% and 36.9%, respectively, in the LRP and RARP group (*p* = 0.56). In cases submitted to eLND, the mean number of lymph nodes removed was 15.16 ± 7.83 and 19.83 ± 4.78, respectively, in LRP and RARP procedures (*p* < 0.001). Mean operative time was 173.33 ± 44.3 min and 153.21 ± 25.1 min, respectively, in LRP and RARP (*p* < 0.001) (Table 1).

#### 3.1.1. Pathological Outcomes

**Figure 1:**
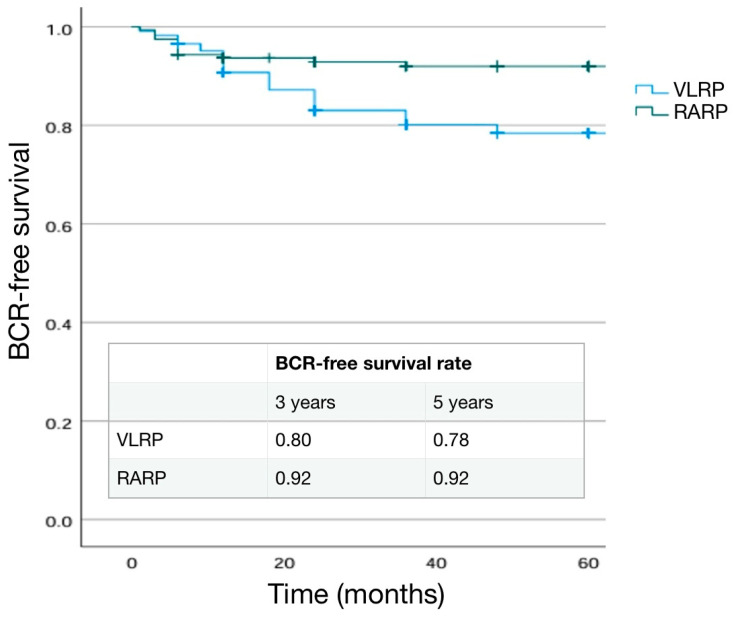
Kaplan–Meier analysis. Estimated rates of biochemical-free survival (BFS) according to the surgical approach. LRP (Laparoscopic) versus robotic-assisted (RARP).

The distribution of pathological T stage was not significantly different (*p* = 0.910) in the two groups. When an eLND was performed, positive lymph nodes (pN1) were found in 15.8% of LRP patients and 13.6% of RARP patients (*p* = 0.430). Pathological ISUP 2 and 3 were more frequent in the RARP group, while the LRP group had a higher percentage of ISUP 1, 4, and 5 cases (*p* < 0.001), (Table 1). SM positivity was not significantly higher in the RARP group (20.0%) when compared to the LRP group (15.9%) (*p* = 0.145). In cases submitted to LRP, the highest rate of SM+ was at the apex (48.9%) followed by the lateral site (24.4%), whereas in cases submitted to RARP it was at the lateral site (37.5%) followed by the apex (28.1%) (*p* = 0.037). Extra-prostatic extension of a positive SM was 3.45 ± 0.84 mm and 2.63 ± 1.14 mm in the LRP and RARP groups, respectively, (*p* = 0.008). SM+ grading was 4 in 24.4% and 31.3% of cases, respectively, in LRP and RARP procedures (*p* = 0.225) (Table 1). During the postoperative follow-up, a BCR was detected in 14.4% and 7.5% of cases in the LRP and RARP groups, respectively, (*p* = 0.014), with a similar mean time to progression (21.77 ± 26 months and 18.58 ± 13 months, respectively, in LRP and RARP) (*p* = 0.1059. Kaplan–Meier curves describing cumulative biochemical recurrence (BCR) free survival rates according to the surgical approach are shown in Figure 1.

Adjuvant therapies were prescribed in 13.0% (73.0% RT and 27.0% RT + ADT) and 10.0% (62.5% RT and 37.5%RT + ADT) of LRP and RARP cases, respectively, (*p* = 0.358) (Table 1).

#### 3.1.2. Functional Outcomes

Postoperative catheterization time was shorter in RARP cases (mean 9.60 ± 2.0) than in LRP cases (mean 11.53 ± 1.45) (*p* < 0.001) (Table 1). The percentage of postoperative side effects after surgery was similar between the two approaches and is described in Table 1 and Table 2.

Postoperative pelvic floor rehabilitation was used in 19.0% and 21.8% of cases, respectively, in LRP and RARP groups (*p* = 0.106) (Table 1 and Table 2). Postoperative PAD tests were similar at 1- and 12-month intervals (*p* > 0.05) between LRP and RARP procedures, with better results at 3-month intervals using the RARP approach (mean pad weight 75.57 ± 122 g and 14 ± 42 g, respectively, in LRP and RARP (*p* < 0.01)) were described (Table 2). In cases submitted to NS procedures, the IIEF5 mean score was 9.60 ± 3.78 at the 6-month and 10.14 ± 4.77 at the 12-month interval for the LRP group, and 10.25 ± 3.94 and 18.0 ± 3.75 for the RARP group (*p* >0.05). The 12-month percentage of IIEF-5 score between 5 and 10 was 57.3% in LRP and 45.4% in RARP (Table 1 and Table 2).

#### 3.1.3. Main Significant Differences in Results

In the RARP group, it was observed that there was: (1) a higher percentage of NS procedure; (2) a higher mean number of lymph nodes removed; (3) a lower mean operative time; (4) a higher (but not statistically significant) rate of SM+; a lower rate of BCR; a lower catheterization time; a lower mean pad weight at 3- and 6-month interval; and a higher (but not statistically significant) IIEF-5 score at the 12-month interval.

### 3.2. Differences in Pathological, Oncological and Functional Outcomes According to Risk Classes

The distribution of the population according to the risk classes showed 142 cases (32.0%) in the low-, 200 cases (45.0%) in the intermediate-, and 102 cases (23.0%) in the high-risk class. Clinical parameters such as age, BMI, and Charlson Index were not significantly (*p* >0.50) different between the three classes (Table 3).

Mean preoperative PSA significantly increased with increasing risk classes (*p* < 0.001). In cases submitted to mMR, the highest percentages of PIRADS 4 and 5 lesions were found, respectively, in the intermediate- and high-risk groups (*p* < 0.01) (Table 3).

A similar proportion of cases was submitted to LRP versus RARP in the three classes (*p* = 0.850). The percentage of cases submitted to an NS procedure was 48.6%, 27.5%, and 3.0%, respectively, in the low-, intermediate- and high-risk class, whereas the percentage of cases submitted to eLND in the intermediate class (based on a risk >5% at predictive nomogram) was 16.5% and 100% in the high-risk class; *p* < 0.001). Operative time was shorter in low-risk cases (mean 150.50 ± 27.35 min) when compared to intermediate- (mean 155.0 ± 20.06 min) and high-risk cases (mean 183.51 ± 50.71 min) (*p* < 0.001) (Table 3).

#### 3.2.1. Pathological Outcomes

The distribution of pathological ISUP varied greatly between the three groups, with only one patient in the low-risk class with a pathological ISUP of 4–5 and only three patients in the high-risk group with a pathological ISUP of 1 (*p* < 0.01). When an eLND was performed, positive lymph nodes (pN1) were found in a higher percentage of cases in the intermediate-risk class when compared with the high-risk class (26.9% vs. 12.8%; *p* < 0.01) (Table 3). SM positivity was higher in the high-risk group (25.5%) when compared to the intermediate- (19.2%) and low-risk groups (9.2%) (*p* = 0.003). SM+ grading was 4 in 0%, 13.2%, and 61.5% of cases, respectively, in low, intermediate and high-risk cases (*p* < 0.00) (Table 3).

A BCR was detected in 4.2%, 8.5% and 29.4% of cases in the low, intermediate and high risk group, respectively, with a similar mean time to progression (19.0 ± 15.83 months, 23.88 ± 28.22 months and 15.72 ± 24.12 months respectively).Adjuvant therapies were prescribed in 2.1% (100% RT), 7.0% (92.9% RT and 7.1% RT + ADT) and 35.3% (58.3% RT and 41.7%RT + ADT) in low, intermediate and high risk cases, respectively, (*p* < 0.001) (Table 3).

#### 3.2.2. Main Significant Differences in Results

Increasing risk class results in: (1) increases in operative time; (2) increases in SM+ rate, and SM+ grading; (3) increases in BRC rate and uses of adjuvant therapies.

### 3.3. Differences in Pathological, Oncological and Functional Outcomes According to pT Stage

A pathologic extra-prostatic tumor was found in 198 cases (44.9%) with pT3a in 34.0% and pT3b in 10.8% of cases. Preoperative PSA significantly (*p* < 0.001) increased from pT2 to pT3b cases (Table 4).

In cases submitted to mpMRI, the percentage of PIRADS 5 lesions significantly increased with increasing pT stage (*p* = 0.01) (Table 4). At biopsy, the percentages of positive cores, bilateral incidence, and ISUP 4–5 were significantly higher (*p* < 0.001) in the pT3 groups (Table 4). A similar proportion of cases was submitted to both the LRP or RARP approach with similar mean operative times in pT2 and pT3 cases (*p* > 0.05) (Table 4).

#### 3.3.1. Pathological Outcomes

**Figure 2:**
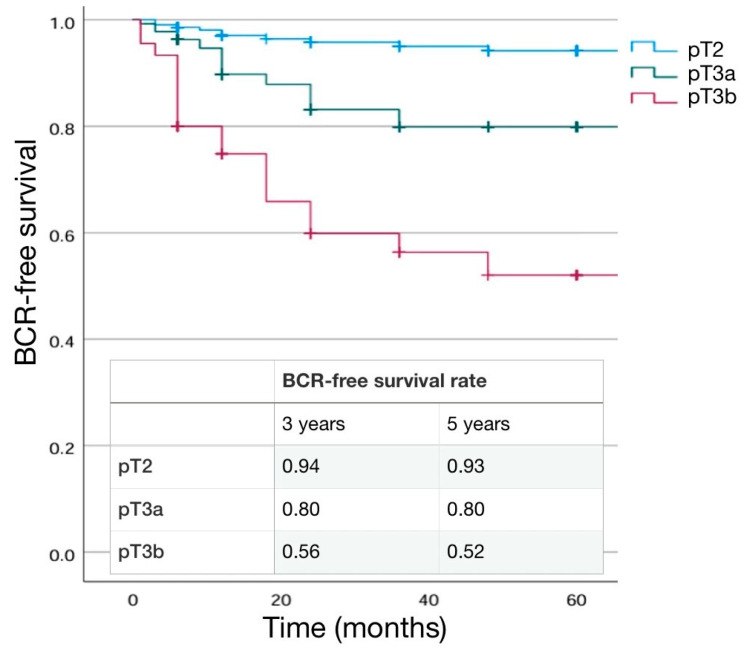
Kaplan–Meier analysis. Estimated rates of biochemical-free survival (BFS) according to the pT stage.

The percentage of ISUP 4–5 PCa at surgery increased with pathological stage (*p* < 0.001), as the percentage of positive lymph node (pN1) (0% in pT2, 11.1% in pT3a and 25.0% in pT3b; *p* < 0.01) (Table 4). SM positivity was higher in the pT3b (37.5%) than in pT3a (20.8%) and pT2 (11.4%) cases (*p* < 0.001). pT3b patients demonstrated a significantly longer mean extra-prostatic extension of SM+ (3.71 ± 1.28) than the other groups (*p* = 0.01), and SM+ grading was 4 in 14.3%, 16.2%, and 66.7% of cases, respectively, in pT2, pT3a and pT3b cases (*p* = 0.001) (Table 4). A BCR was detected in 4.9%, 13.3%, and 37.5% of cases in the pT2, pT3a, and pT3b group, respectively, with a shorter mean time to progression in the pT3b group (34.25 ± 42.2, 16.1 ± 11.3, and 11.28 ± 15.1 in the pT2, pT3a, and pT3b groups, respectively; *p* = 0.032).Adjuvant therapies were prescribed in 1.6% (100% RT), 11.3% (82.4% RT and 17.6% RT + ADT), and 62.7% (59.5% RT and 40.6%RT + ADT) in pT2, pT3a, and pT3b cases, respectively (*p* < 0.001) (Table 4). Kaplan–Meier curves describing cumulative biochemical recurrence (BCR) free survival rates according to pT stage are shown in Figure 2.

#### 3.3.2. Main Significant Differences in Results

Increasing pT stage leads to an: (1) increase in preoperative PSA; (2) increase in PIRADS 5 lesions and percentage of positive biopsy cores; (3) increased rate of positive lymph nodes (pN+), SM+; increase in SM+ grading, BCR rate, and the use of adjuvant therapies.

### 3.4. Differences in Pathological, Oncological and Functional Outcomes According to pN Stage

A low percentage (14.8%) of cases submitted to eLND showed pathological lymph node involvement, with a similar distribution between the LRP (15.8%) and RARP (13.6%) approach. Clinical parameters such as age, BMI, and Charlson index were not significantly (*p* > 0.50) different between pN0 and pN1 cases (Table 5).

Mean total PSA was significantly higher in pN1 patients (16.54 ± 8.83 vs. 8.72 ± 5.29 in the pN1 and pN0 groups, respectively; *p* < 0.001) (Table 5). A clinical diagnosis suspicious for lymph node involvement at preoperative imaging was found in 0.9% of cases in pN0 and 15.5% of cases in pN1 cases (*p* < 0.001). At biopsy, the percentage of positive cores and bilateral incidence were significantly higher (*p* = 0.001) in the pN1 group. The distribution of ISUP grading (*p* = 0.304) and mean and median nomograms results were similar between pN0 and pN1 groups (*p* > 0.05) (Table 5).

#### 3.4.1. Pathological Outcomes

**Figure 3:**
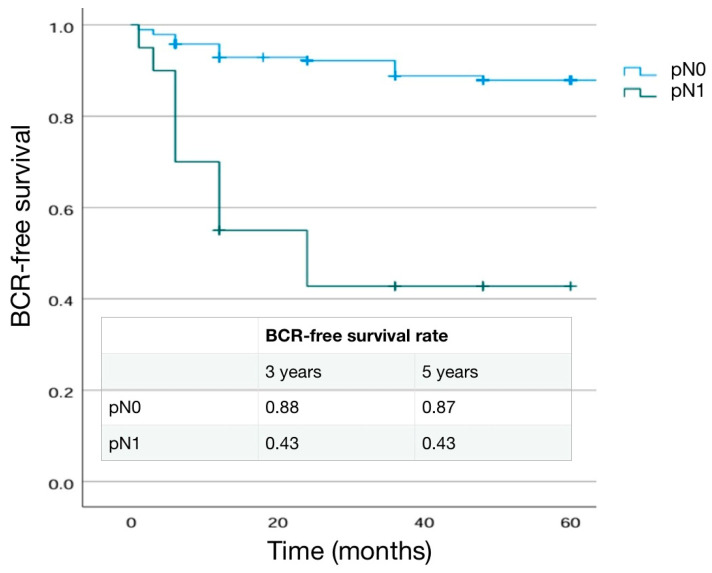
Kaplan–Meier analysis. Estimated rate of biochemical-free survival (BFS) according to the pN stage.

The mean number of lymph nodes removed was similar in the two groups (*p* = 0.60). In pN1 cases the site of positive lymph nodes (pN+) was obturatory (100%), internal iliac (45.0%), and esternal iliac (40.0%) (Table 5). The percentage of pathological ISUP 5, pT3b stage, and cribiform differentiation were significantly higher in pN1 patients (*p* = 0.001, 0.001 and <0.001, respectively) (Table 5). Also SM positivity was higher in the pN1group (45.0%) than in pN0 (27.9%) cases (*p* = 0.001) and SM+ grading was 4 in 37.5% and 55.6% of cases, respectively, in pN0 and pN1 cases (*p* = 0.384) (Table 5). A BCR was detected in 18.3% and 55.0% of cases in the pN0 and pN1 group, respectively (*p* < 0.001), with a shorter mean time to progression in the pN1 group (4.18 ± 7.37 months and 28.3 ± 34.6 months, respectively, in pN1 and pN0). Adjuvant therapies were prescribed in 26.1% (80% RT and 20% RT + ADT) and 70.0% (28.6% RT and 71.4%RT + ADT) of pN0 and pN1 cases, respectively (*p* < 0.001) (Table 5). Kaplan–Meier curves describing cumulative biochemical recurrence (BCR) free survival rates according to the pN stage are shown in Figure 3.

#### 3.4.2. Main Significant Differences in Results

In pN1 cases, there was observed to be a: (1) higher clinical suspicious cN1; higher preoperative PSA and percentage of positive core at biopsy; (2) higher pT3b rate, ISUP 5 rate, SM+ rate and SM+ grading; (3) higher BCR rate and use of adjuvant therapies.

### 3.5. Differences in Pathological, Oncological and Functional Outcomes According to Surgical Margins

Positive surgical margins were found in 17.3% of cases with no significant difference between the LRP (15.9%) and the RARP (20.0%) group (*p* = 0.145). Clinical parameters such as age, Charlson Index, and BMI were not significantly (*p* > 0.50) different between SM− and SM+ cases (Table 6).

The SM+ group had a significantly higher mean preoperative PSA (11.54 ± 8.8 vs. 8 ± 1.07; *p* < 0.001). A higher percentage of clinical stage cT1, cT2a, and cT2b in the SM− group and a higher percentage of cT2c, cT3a, and cT3b cases in the SM+ group was present (*p* < 0.001) (Table 6). At biopsy, the percentage of positive cores was significantly higher (*p* < 0.001) in the SM+ group. The distribution of ISUP grading was similar between SM− and SM+ groups (*p* = 0.14) (Table 6).

The percentage of cases submitted to an NS procedure was 29.6% and 25.3%, respectively (*p* = 0.450), and mean operative time was 160.03 ± 36.02 min and 160.63 ± 22.39 min, respectively, in SM− and SM+ (*p* = 0.92) (Table 6).

#### 3.5.1. Pathological Outcomes

**Figure 4:**
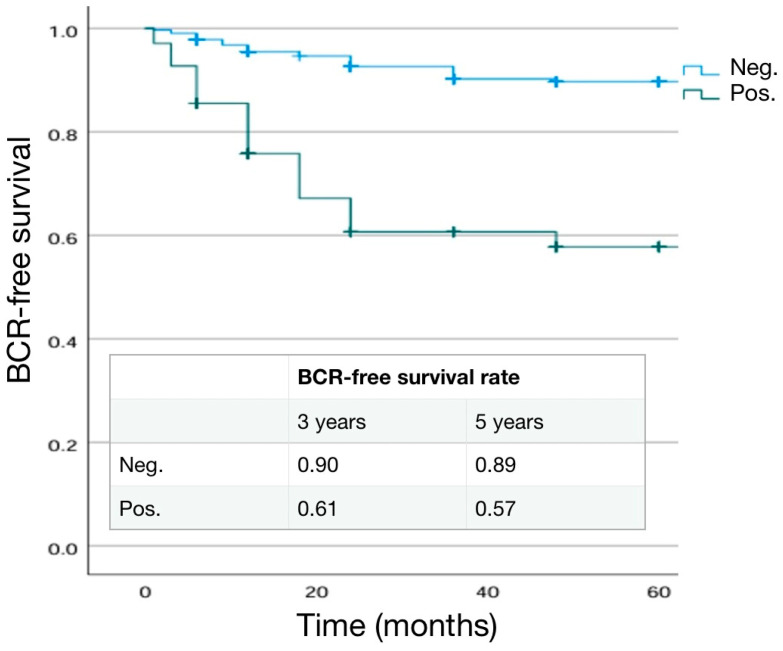
Kaplan–Meier analysis. Estimated rates of biochemical-free survival (BFS) according to surgical margin (SM) status.

The distribution of pathological ISUP grading and T stage were significantly different (*p* = 0.006 and <0.001 respectively) in the two groups. In particular, pT3 cases were more frequent in SM+ patients than in SM− patients (63.6% and 40.6%) (Table 6). The site for positive SM was posterior–lateral in 45.5% of cases, basal in 7.8%, and apex in 40.2% of cases. Median extra-prostatic radial extension of positive SM was 3 mm (range 1–7) and SM+ grading was 4 in 27.3% and 3 in 71.4% of cases (Table 6). A BCR was detected in 7.9% and 31.2% of SM− and SM+ cases, respectively, with a shorter mean time to progression in SM+ cases (25.45 ± 29.4 months and 9.73 ± 11.57 months, respectively, in SM− and SM+). Adjuvant therapies were prescribed in 8.4% (40% RT and 60% RT + ADT) and 55.9% (76.7% RT and 23.3%RT + ADT) in SM− and SM+ cases, respectively (*p* < 0.001) (Table 6). Kaplan–Meier curves describing cumulative biochemical recurrence (BCR)-free survival rates according to the SM status are shown in Figure 4.

#### 3.5.2. Main Significant Differences in Results

In SM+, there was a: (1) higher preoperative PSA; (2) higher percentage of positive core at biopsy; increase in BCR rate; increase in adjuvant therapy use

### 3.6. Differences in Pathological, Oncological and Functional Outcomes According to Biochemical Recurrence

Biochemical recurrence (BCR) was evaluated at a mean postoperative follow up of 56.4 ± 37.8 years. A BCR was detected in 11.9% of cases with a mean time of 18.67 ± 24.57 months, with a lower percentage in cases submitted to RARP (7.5%) than in cases submitted to LRP (14.4%) (*p* = 0.014).

Clinical parameters such as age and Charson Index were not significantly (*p* > 0.50) different between BCR− and BCR+ cases (Table 7).

On the contrary, BCR+ patients had a significantly higher preoperative mean BMI (27.21 ± 3.61 vs. 25.74 ± 3.43; *p* = 0.017) (Table 7). At biopsy the percentage of positive cores and bilateral incidence were significantly higher (*p* < 0.001) in the BCR+ group. The distribution of ISUP grading also varied significantly between the two groups. In fact, only 2% of patients that did not develop a BCR were found to have an ISUP of 5 at biopsy, while 15.1% of those who experienced a BCR had an ISUP grade of 5 at diagnostic biopsy (*p* < 0.001) (Table 7).

The percentage of cases submitted to a NS procedure was 30.9% and 11.3%, respectively, in the BCR− and BCR+ group (*p* = 0.002) (Table 7).

#### 3.6.1. Pathological Outcomes

**Figure 5:**
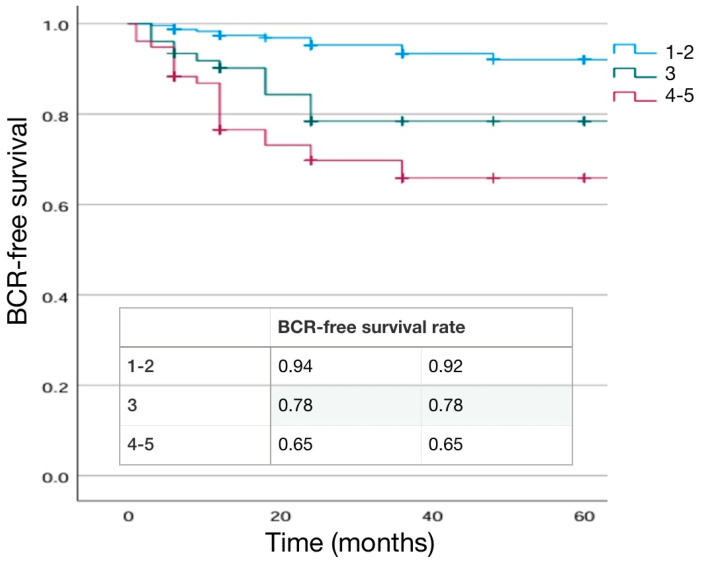
Kaplan–Meier analysis. Estimated rates of biochemical-free survival (BFS) according to the ISUP grading at surgery.

As expected, BCR+ patients were more likely to have a higher pathological stage and ISUP grading at final pathology (*p* < 0.001). Similarly, positive lymph nodes (pN1) in the case of an eLND were found in a higher percentage of cases in the BCR+ group (8.7% in BCR− and 34.4% in BCR+; *p* = 0.014) (Table 7). SM positivity was higher in the BCR+ (45.3%) than in BCR− (13.6%) cases (*p* < 0.001). Mean extra-prostatic extension of positive SM was higher in BCR+ patients (3.75 ± 1.35 mm vs. 2.67 ± 0.8 mm; *p* = 0.001) and SM+ grading was 4 in 13.2% and 58.3% of cases, respectively, in BCR− and BCR+ cases (*p* < 0.001) (Table 7). Kaplan–Meier curves describing cumulative biochemical recurrence-free survival rates according to the different variables are shown in Figure 1, Figure 2, Figure 3, Figure 4 and Figure 5.

#### 3.6.2. Main Significant Differences in Results

In BCR+, there was a: (1) higher preoperative PSA; (2) higher percentage of positive prostatic core at biopsy; (3) higher pT stage and ISUP grading; (4) higher percentage of SM+ and SM+ grading.

### 3.7. Logistic Regression Analysis

Table 8 shows a logistic regression analysis assessed to identify variables able to condition adverse pathological, oncological, and functional conditions in our population of non-metastatic PCs submitted to surgery.

#### 3.7.1. Predictors for the Risk of Extracapsular Extension

Upon univariate analysis, the risk of extracapsular extension at pathological staging after surgery did not significantly vary according to percentage of positive tissue per core at biopsy and biopsy laterality, whereas it significantly increased according to preoperative PSA (OR= 3.46; 95% CI= 2.19–5.44; *p* < 0.002), PIRADS score (highest OR = 4.84; 95% CI = 1.97–11.93; *p* < 0.001), ISUP grading (highest OR = 10.63; 95% CI 5.79–19.52; *p* < 0.001), and risk class (high risk: OR = 2.9; 95% CI = 1.74–4.89; *p* < 0.001). Upon multivariate analysis, ISUP grading 4–5 and high-risk class were the only variables able to independently and significantly influence the risk for extracapsular extension (*p* = 0.006 and *p* = 0.04 respectively) (Table 8a).

#### 3.7.2. Predictors for the Risk of Upgrading at Surgery

Upon univariate analysis, the risk of upgrading after surgery (ISUP 1–2 in ISUP 3–5) did not significantly vary according to PIRADS score at mpMRI and percentage of positive tissue per core. Considering the intermediate-risk class as standard reference, the risk of upgrading significantly increased 2.9 times (95%CI 1.74–4.89; *p* < 0.001) in cases with a high-risk tumor. On multivariate analysis, only high-risk class remains a variable able to independently and significantly influence the risk for upgrading (*p* = 0.04) (Table 8b).

#### 3.7.3. Predictors for the Risk of Lymph Node Involvement

On univariate analysis, the risk of lymph node involvement at pathological evaluation (pN1) did not significantly vary according to the percentage of positive tissue per core biopsy, Briganti 2019 nomogram risk using a 7% cut-off (OR = 1.3; 95% CI = 1.07–1.49, *p* = 1.61), and surgical approach (OR = 1.2; 95%CI= 0.5–3.12; *p* = 0.60). Upon univariate analysis, variables able to significantly increase the risk of lymph node involvement were preoperative PSA with a >15 ng/mL value (OR = 4.27; 95%CI 1.65–11.02; *p* = 0.001), high-risk class (OR 7.32; 95%CI 2.06–25.94, *p* < 0.001), number of lymph nodes removed at surgery >15 vs. <10 (OR 5.69; 95%CI = 1.78–18.17; *p* = 0.001), ISUP grading 3 vs. 1–2 and 4–5 vs. 1–2 (OR = 9.45; 95%CI = 1.93–46.32; *p* = 0.003 and OR = 9.28; 95%CI = 1.96–43.86; *p* < 0.001 respectively), and pT stage, pT3a vs. pT2 and pT3b vs. pT2 (OR= 1.08; 95%CI = 1.01–1.15; *p* = 0.012 and OR = 1.7; 95%CI = 1.28–2.25; *p* < 0.001).

Upon multivariate analysis, a PIRADS score of 4 vs. 3 and 5 vs. 3 (*p* = 0.01 and *p* = 0.03), high-risk class (0.002), an ISUP grading of 3 vs. 1–2 and 4–5 vs. 1–2 (*p* = 0.005 and *p* < 0.003), pT3b vs. pT2 (*p* = 0.003), and the number of removed lymph nodes >15 versus <10 (*p* = 0.049) were the variables able to independently and significantly influence the risk for lymph node involvement (Table 8c).

#### 3.7.4. Predictors for the Risk of Positive Surgical Margins

Upon univariate analysis, the risk of positive surgical margins at pathological evaluation did not significantly vary according to prostate volume, positive biopsy laterality, risk class, NS technique (OR = 0.8; 95%CI = 0.46–1.44; *p* = 0.48), surgical approach (OR = 1.27; 95%CI = 0.7–2.1; *p* = 0.35), or operative time >120 min vs. <120 min (OR = 1.57; 95%CI 0.34–7.27.; *p* = 0.56). On the contrary, the risk of SM+ increased with preoperative PSA >10 ng/dL vs. <10 ng/dL (OR = 3.57; 95%CI = 1–5.63; *p* < 0.001), a PIRADS of score 5 vs. 3 (OR3.32; 95%CI 1.08–10.23; *p* = 0.003), pT3a vs. pT2 and pT3b vs. pT2 (OR = 2; 95%CI 1.15–3.52; *p* = 0.01 and OR = 4.6; 95%CI 2.27–9.32; *p* < 0.001), and ISUP grading of 4–5 vs. 1–2 (OR = 1.84; 95%CI 1–3.41; *p* = 0.05).

Upon multivariate analysis, only preoperative PSA and pT3b vs. pT2 remained variables able to independently and significantly influence the risk for SM+ (*p* = 0.04 and *p* < 0.001, respectively) (Table 8d).

#### 3.7.5. Predictors for the Risk of Biochemical Recurrence

Upon univariate analysis, the risk of biochemical recurrence (BCR) during the postoperative follow-up was significantly related to several variables. In particular, the risk for BCR increased with preoperative PSA >10 ng/mL vs. <10 ng/mL (OR = 3; 95%CI 1.68–5.57; *p* < 0.001), a PIRADS of score 5 vs. 3 (OR= 1.42; 95%CI 1.17–1.71; *p* < 0.001), positive biopsy laterality (OR= 2.64; 95%CI= 1.37–5.1; *p* = 0.003), risk class (OR = 4.35; 95%CI 2.47–8.44; *p* < 0.001), pT3a vs. pT2 and pT3b vs. pT2 (OR = 3.42; 95%CI 1.64–7.14; *p* < 0.001 and OR = 11.1; 95%CI 4.8–25.75; *p* < 0.001 respectively), an ISUP grading of 3 vs. 1–2 and 4–5 vs. 1–2 (OR = 3.21; 95%CI 1.47–7; *p* = 0.005 and OR = 6.76; 95%CI 3.32–13.7; *p* < 0.001), number of lymph nodes removed >15 vs. <10 (OR = 2.86; 95%CI 1.24–6.6; *p* = 0.01), SM positivity (OR = 4.97; 95%CI 2.39–10.34; *p* < 0.001), grading at SM+ 4 vs. 3 (OR = 50; 95%CI 5.57–451; *p* < 0.001), and PNI positivity at final pathology (OR 4.5; 95%CI 1.92–10.55; *p* < 0.001). Finally, the risk for BCR was lower in patients who underwent a RARP when compared to a LRP and in those submitted to an NS technique (OR = 0.38; 95%CI = 0.19–0.75; *p* = 0.007 and OR = 0.24; 95%CI 0.09–0.62; *p* = 0.002).

Upon multivariate analysis, PSA > 10 ng/mL (*p* = 0.007), high risk class (*p* = 0.007), ISUP 4–5 (*p* = 0.006), lymph node involvement (*p* = 0.014), SM positivity (*p* = 0.004), and an SM+ grading of 4 (*p* < 0.001) remained significant and independent predictors for the development of a BCR (Table 8e)

## 4. Discussion

Radical prostatectomy remains a primary treatment for non-metastatic prostate cancer, with minimally invasive techniques like laparoscopic radical prostatectomy and robotic-assisted radical prostatectomy being widely adopted. As these techniques continue to evolve, updated evidence is critical for understanding their comparative effectiveness, safety, and patient outcomes [26,27,28,29,30].

LRP, although effective, requires significant technical expertise. Surgeons must develop advanced laparoscopic skills, particularly in high-volume centers, to achieve outcomes that match those of RARP [31,32,33,34,35,36]. A study by Bhayani and Pavlovich [1] reaffirms the steep learning curve associated with LRP, emphasizing the need for extensive training and experience to achieve optimal patient outcomes. Stolzenburg JU [2] reported that while LRP remains effective in experienced hands, the advantages of RARP in preserving functional outcomes, particularly continence, are becoming more pronounced with robotic technology.

RARP, in some studies, has been associated with lower positive surgical margin rates, particularly in high-risk prostate cancer cases, and better preservation of urinary continence and sexual function due to enhanced nerve-sparing capabilities [12,14,15,16,20,21,22]. A recent systematic review by Ma J et al. [3] highlighted the technological superiority of RARP in terms of precision and ergonomics, contributing to better surgical outcomes and shorter learning curves for surgeons compared to LRP. A meta-analysis by Huang X et al. [4] found that RARP had significantly better functional outcomes than LRP, especially in terms of early continence recovery and sexual function preservation, making it the preferred choice in several centers. A multi-institutional study by Dell’Oglio et al. [5] confirmed that RARP continues to demonstrate superior functional outcomes compared to LRP, with lower rates of postoperative complications.

The latest guidelines by the European Association of Urology reflect the growing preference for RARP in centers where it is available, citing its advantages in functional outcomes. However, LRP remains a recommended alternative, and the EAU Guidelines on Prostate Cancer (2024) emphasize that both LRP and RARP are viable options, with the choice depending on the availability of technology and surgeon expertise [7]. RARP may be favored for its superior functional outcomes, particularly in terms of continence and potency preservation [12,14,15,16,20,21,22]. The decision between these techniques should be individualized based on patient characteristics, surgeon experience, and available resources, in line with the latest European guidelines [7].

In the present study we report on the long-term case history of a population of patients with non-metastatic PCa considered suitable, according to EAU guidelines and after multidisciplinary discussion, for radical prostatectomy, performed by a single primary surgeon with experience of over 10 years with LRP and over 5 years with RARP. This population represents a homogeneously treated sample in a perfect real-life situation at a high-volume institution for the treatment of PCa and with a multidisciplinary (MDT) group regulating decisions relating to the management of each individual patient. *Limitations and strengths:* Strengths of the study are the analysis of a real-world situation in a high-volume center and with an MDT group that determines homogeneous evaluation and treatment criteria. Furthermore, as a university training center, all LRP and RARP procedures were performed by a single surgeon with an operating team of urology residents. The limitations are mainly the lack of randomization in the selection of patients for the two procedures (LRP versus RARP). No selection parameters were considered to determine the surgical procedure; as a result, the non-randomization produced some differences between the population undergoing LRP and that undergoing RARP. In particular, age was significantly higher in the LRP group, whereas the percentage of PIRADS 4–5 at mpMRI and the percentage of clinical locally advanced stage (cT3) was significantly higher in the RARP group. Except for these parameters, the two populations are sufficiently homogeneous at baseline.

In the comparison between the robotic and laparoscopic approach, a first advantage of the robotic approach is a significant reduction in operating times, days of hospitalization and postoperative catheterization compared to laparoscopic surgery (despite the greater experience of the operator >10 years in LRP compared to robotics >5 years). Perioperative and postoperative complications such as anastomotic leakage, need of blood transfusion, and urethral stricture have practically disappeared with both approaches. Both approaches have similarly resulted in a drastic reduction in postoperative complications in clinical practice; the greater simplicity of the robotic approach also produces an advantage in terms of operating, catheterization, and hospitalization times.

With a homogeneous indication for extended lymph node dissection (all high-risk cases and >5% risk at Briganti nomogram in intermediate risk), a similar percentage of cases was submitted to eLND using RARP or LRP. RARP was able to remove a significantly higher number of lymph nodes, however the percentage of pN1 was similar and limited in both approaches (LRP 15.8%, RARP 13.6%). The role of extended lymphadenectomy in RP remains debatable due to the low percentages of pN1 reported in most experiences, regardless of the number of lymph nodes removed at surgery and despite indications obtained by nomograms [13]. In our experience, the multivariate analysis, together with PIRADS score, high risk class, ISUP grading, and pT stage, as well as the number of removed lymph nodes being >15 versus <10, was a variable able to independently and significantly influence the risk for pathologic lymph node involvement (OR 4.10; 95%CI 1.0–26.5; *p* = 0.049), whereas surgical approach was not a significant variable.

Our experience did not confirm significant differences in terms of risk for SM+ related to the surgical approach although the percentage was slightly higher after RARP. A higher but not statistically significant percentage of positive surgical margins was found after RARP when compared to LRP, but with lower extra-prostatic radial distance in the RARP group. At the multivariate analysis only a pathological involvement of seminal vesicles (pT3b stage) was able to significantly and independently increase the risk of SM+.

The only parameter significantly influenced by the surgical approach in our population was biochemical recurrence at postoperative follow-up. The RARP approach was associated with a significantly lower percentage of BCR (7.5%) when compared to LRP (14.4%) (*p* = 0.014). Time to biochemical progression was similar between LRP and RARP cases but in the RARP group the risk for BCR was significantly reduced at univariate analysis. However, at multivariate analysis the surgical approach did not remain a significant and independent variable able to influence BCR rates, as opposed to surgical margins positivity and grade, lymph node involvement and ISUP grading.

The second crucial point in the comparative analysis between RARP and LRP, is the possibility of a functional long-term advantage. In our experience, the percentage of nerve sparing techniques was double in the RARP group when compared to the LRP. Robotic vision probably determines greater safety in performing a nerve sparing approach, leading to an extension of the indications in clinical practice. In our clinical practice we extensively use pelvic floor rehabilitation with electrostimulation and biofeedback starting 30 days after removal of the catheter, homogeneously in RARP (21.8%) and LRP (19.0%) cases. RARP was capable of speeding up the reduction of postoperative urinary losses with a significantly lower PAD weight at 3 and 6 months postoperatively compared to LR (mean pad weight 75.57 ± 122 g and 14 ± 42 g, respectively, in LRP and RARP (*p* < 0.01)) while at 12 months the recovery of continence was high in both approaches (no PADS in 91.6% of cases). In cases submitted to a nerve sparing procedure, the two approaches produced no statistically significant differences in terms of IIEF-5 score reduction at 6 and 12 months postoperatively (*p* > 0.05). However, the 12-month IIEF-5 mean and median scores showed better results using RARP (18.0 ± 3.75; 18) than LRP (10.14 ± 4.77; 8). Considering ED as a postoperative International Index of Erectile Function-5 (IIEF-5) score between 5 and 10, the 12-month percentage of ED was 57.3% in LRP and 45.4% in RARP.

## 5. Conclusions

In the comparison between the robotic and laparoscopic approach, a clear advantage of the robotic approach is a significant reduction in operating times, days of hospitalization, and postoperative catheterization compared to laparoscopic surgery. Perioperative and postoperative complications such as anastomotic leakage, need of blood transfusion, and urethral stricture have practically disappeared with both approaches. It is not possible to describe any certain oncological advantage both in terms of surgical margins and pathological lymph nodes removed. A reduction to the limit of significance is described in terms of biochemical recurrence to the advantage of the robotic approach. RARP produces a more rapid recovery of urinary continence at 3 months postoperatively without significant advantages in terms of erective potency recovery.

## 6. Patents

The choice of a robotic-assisted approach for radical prostatectomy procedure can provide a clear advantage in terms of reduction of operating time, days of hospitalization and catheterization compared to the laparoscopic surgery. Perioperative complications such as anastomotic leakage, need of transfusion, and urethral stricture have practically disappeared with both approaches. Regarding oncological results, a reduction to the limit of significance in terms of biochemical recurrence rate to the advantage of the robotic approach was observed. The robotic approach produces a more rapid recovery of urinary continence at 3 months postoperatively without significant advantages in terms of erective potency recovery.

## Figures and Tables

**Table 1:** Distribution of preoperative and postoperative parameters on the basis of surgical approach. Mean ± SD, median, (range). Number of cases (%) (LRP = laparoscopic; RARP = robotic-assisted).

	LRP	RARP	*p* Value
Number cases	284	160	
Age (years)	68.65 ± 6.582; 69: (48–73)	66.35 ± 5.973; 66 (47–68)	<0.001
BMI	26.17 ± 11.16; 25.6: (17–39.4)	25.127 ± 4.317; 25.0 (17–34)	0.124
Charlson Index	3.34 ± 1.42; 4 (2–7)	4.10 ± 0.722; 4 (0–7)	<0.001
Familiarity			0.217
- Yes	25 (8.8%)	10 (6.2%)	
- No	259 (91.2%)	150 (93.8%)	
Digital Rectal Examination			0.327
- Normal	245 (86.3%)	131 (81.9%)	
- Suspicious	39 (13.7%)	29 (18.1%)	
Preoperative total PSA (ng/mL)	8.79 ± 6.175; 7.5: (3.0–64.0)	8.23 ± 4.89; 6.9 (3.0–30.0)	0.328
PSAD	0.16 ± 0.10; 0.14: (0.04–0.59)	0.22 ± 0.18; 0.17 (0.07–0.59)	0.546
Prostate volume (cc)	49.67 ± 16.07; 49.5: (22–120)	47.56 ± 14.39; 45 (20–120)	0.409
**mMR PIRADS score**			0.042
(data available in 208 cases)			
PIRADS 2	3 (4.3%)	3 (2.2%)	
PIRADS 3	21 (30.0%)	23 (16.7%)	
PIRADS 4	33 (47.1%)	78 (56.5%)	
PIRADS 5	13 (18.6%)	34 (24.6%)	
Prostate Tumor size (mm) at mMR	10.76 ± 5.11; 10.0: (6–39)	13.59 ± 6.94; 12.0 (4–38)	0.10
Preoperative CT and bone scan			0.251
- No	245 (86.3%)	129 (80.6%)	
- Yes	39 (13.7%)	31 (19.4%)	
Preoperative PET CT scan			0.117
- No	283 (99.6%)	151 (94.3%)	
- Choline	1 (0.4%)	5 (3.2%)	
- PSMA	0	4 (2.5%)	
**Clinical T staging**			<0.001
T1	9 (3.2)	3 (1.9%)	
T2a	19 (6.7%)	11 (6.9%)	
T2b	110 (38.7%)	82 (51.2%)	
T2c	131 (46.1%)	42 (26.2%)	
T3a	12 (4.2%)	18 (11.3%)	
T3b	3 (1.1%)	4 (2.5%)	
**Clinical N staging**			0.545
N0	282 (99.3%)	158 (98.7%)	
N1	2 (0.7%)	2 (1.3%)	
Number of suspected lymph nodes at imaging	4 ± 1.4; 4: (3–4)	2 ± 1.35; 2 (1–3)	0.64
**Biopsy outcomes**			
% positive samples PCa	40.6 ± 26.26; 29: (8–100)	41.60 ± 26.09; 35 (4–57)	0.772
Prostate biopsy laterality +			<0.001
- Monoliteral	102 (35.9%)	101 (63.1%)	
- Bilateral	182 (64.1%)	59 (36.9%)	
**ISUP grading at biopsy**			0.610
1	98 (34.5%)	51 (31.9%)	
2	74 (26.1%)	61 (38.1%)	
3	58 (20.4%)	28 (17.5%)	
4	42 (14.8%)	15 (9.4%)	
5	12 (4.2%)	5 (3.1%)	
**Risk Class (D’Amico)**			0.849
- Low risk	92 (32.4%)	50 (31.3%)	
- Intermediate risk	126 (44.3%)	74 (46.2%)	
- High risk	66 (23.3%)	36 (22.5%)	
**Operative time (minutes)**	173.33 ± 44.34; 160 (90–300)	153.21 ± 25.11; 142.5 (90–300)	<0.001
**Nerve sparing technique at surgery**			<0.001
-** No**	223 (78.5%)	94 (58.7%)	
-** Yes**	61 (21.5%)	66 (41.3%)	
- Monolateral	27 (43.3%)	25 (31.7%)	
- Bilateral	34 (56.7%)	41 (68.3%)	0.187
**Extended lymph node dissection**			0.160
-** No**	146 (73.2%)	74 (63.1%)	
-** Yes**	76 (26.8%)	59 (36.9%)	
**Pathological stage (T)**			0.910
pT2	157 (55.3%)	89 (55.6%)	
pT3a	91 (32.0%)	59 (36.9%)	
pT3b	36 (12.7%)	12 (7.5%)	
pT4	0	0	
**Pathological stage (N)**			0.430
N0	64(84.2%)	51 (86.4%)	
N+	12(15.8%)	8 (13.6%)	
**Number Lymph nodes removed at surgery**			<0.001
- Total cases	15.16 ± 7.83; 15: (4–47)	19.83 ± 4.78; 20 (6–34)	
- N+ cases	17.45 ± 11.8; 15 (6–47)	19.6 ± 7.1; 20 (6–34)	
- N0 cases	14.7 ± 6.8; 14 (4–35)	19.8 ± 4.1; 20 (11–31)	
**ISUP grading at surgery**			<0.001
1	73 (25.7%)	19 (11.9%)	
2	98 (34.5%)	85 (53.1%)	
3	54 (19.0%)	35 (21.9%)	
4	37 (13.0%)	11 (6.9%)	
5	22 (7.8%)	10 (6.2%)	
**Surgical margin at surgery (R)**			0.145
- Negative	239 (84.1%)	128 (80.0%)	
- positive	45 (15.9%)	32 (20.0%)	
**Positive surgical margin site**			0.037
-** Apex**	22 (48.9%)	9 (28.1%)	
-** Lateral**	11 (24.4%)	12 (37.5%)	
-** Basal**	2 (4.4%)	4 (12.5%)	
-** Posterior**	7 (15.6%)	5 (15.6%)	
-** Multiple**	3 (6.7%)	2 (6.3%)	
**Positive surgical margin grading**			0.225
-** 3**	34 (75.6%)	21 (65.6%)	
-** 4**	11 (24.4%)	10 (31.3%)	
-** 5**	0 (0%)	1 (3.1%)	
**Positive surgical margin radial distance (mm)**	3.45 ± 0.84; 3 (2–6)	2.63 ± 1.14; 2.5 (1–7)	0.008
-** Positive surgical margin**			0.762
-** Single**	42 (93.3%)	30 (93.7%)	
-** Multiple**	3 (6.7%)	2 (6.3%)	
**PNI at surgery**			0.10
- Positive	179 (62.6%)	90 (56.2%)	
- Negative	105 (37.4%)	70 (43.8%)	
**Cribriform/IDC at surgery**			0.01
- Positive	7 (2.5%)	13 (8.1%)	
- Negative	277 (97.5%)	147 (91.9%)	
**Postoperative hospitalization (days)**	3.75 ± 0.74; 3 (3–7)	3.11 ± 0.36; 3 (3–5)	0.037
**Catheterization time (days)**	11.53 ± 1.45; 12 (8–14)	9.60 ± 2.06; 10 (6–16)	<0.001
**Postoperative anastomotic leakage**			0.245
-** No**	284 (100%)	157 (98.1%)	
-** Yes**	0 (0%)	3 (1.9%)	
**Postoperative blood transfusion**			0.294
-** No**	284 (100%)	158 (98.7%)	
-** Yes**	0 (0%)	2 (1.3%)	
**Postoperative lymphocele**			0.16
-** No**	278 (97.9%)	158 (98.7%)	
-** Yes**	6 (2.1%)	2 (1.3%)	
**Postoperative anastomotic stricture**			0.51
-** No**	272 (95.8%)	160 (100%)	
-** Yes**	12 (4.2%)	0 (0%)	
**Postoperative rectal injury**			--
-** No**	284 (100%)	160 (100%)	
-** Yes**	0	0	
Postoperative total PSA (ng/mL)(at 1 month)	0.06 ± 0.2; 0.02: (0.01–2.0)	0.04 ± 0.92; 0.02 (0.01–1.0)	0.047
Postoperative follow-up (years)	64.4 ± 28.8; 62 (24–120)	49.2 ± 36.0; 48 (12–120)	0.385
Biochemical progression			0.014
No	243 (85.6%)	148 (92.5%)	
Yes	41 (14.4%)	12 (7.5%)	
Time to biochemical progression (months)	21.77 ± 26.49; 12 (1–120)	18.58 ± 13.2; 13 (1–36)	0.105
Adjuvant therapy			0.358
- No	247 (87.0%)	143 (90.0%)	
- Yes	37 (13.0%)	16 (10.0%)	
Adjuvant therapy type			0.358
- RT	27 (73.0%)	10 (62.5%)	
- RT + ADT	10 (27.0%)	6 (37.5%)	
IIEF-5 postoperative (6 months) (patients submitted to nerve sparing)	9.60 ± 3.78; 9 (6–19)	10.25 ± 3.94; 9.5 (7–15)	0.750
IIEF-5 postoperative (12 months) (patients submitted to nerve sparing)	10.14 ± 4.77; 10 (5–21)	18.0 ± 3.75; 18 (8–18)	0.117
Postoperative Pelvic floor rehabilitation			0.106
- No	230 (81.0%)	125 (78.2%)	
- Yes	54 (19.0%)	35 (21.8%)	
**Postoperative PAD test**			
1 months (grams)	163.84 ± 222.0; 50 (0–400)	68.17 ± 374.17; 11 (0–404)	0.680
3 months (grams)	75.57 ± 122.20; 20 (0–480)	14.02 ± 42.09; 0 (0–250)	<0.001
6 months (grams)	39.47 ± 76.08; 5 (0–420)	13.62 ± 28.60; 0 (0–80)	0.023
12 months (grams)	14.76 ± 29.41; 0 (0–100)	15.33 ± 23.2; 1 (0–50)	0.964

**Table 2:** (**a**) Postoperative side effects according to the surgical approach (LRP = Laparoscopic versus RARP = Robotic-assisted). Number of cases and % of cases. Mean ± SD, median, (range). (**b**) Postoperative urinary continence results according to the surgical approach (LRP = Laparoscopic versus RARP = Robotic-assisted). Number of cases and % of cases and mean ± SD, median and range. (**c**) Postoperative sexual recovery results according to the surgical approach (LRP = Laparoscopic versus RARP = Robotic-assisted). Number of cases and % of cases and mean ± SD, median and range.

**(a)**			
**Parameter**	**LRP**	**RARP**	***p* Value**
**Postoperative anastomotic leakage**			0.245
-** No**	284(100%)	157 (98.1%)	
-** Yes**	0	3 (1.9%)	
**Postoperative blood transfusion**			0.294
-** No**	284 (100%)	158 (98.7%)	
-** Yes**	0	2 (1.3%)	
**Postoperative lymphocele**			0.16
-** No**	278 (97.9%)	158 (98.7%)	
-** Yes**	6 (2.1%)	2 (1.3%)	
**Postoperative anastomotic stricture**			0.51
-** No**	272 (95.8%)	160 (100%)	
-** Yes**	12 (4.2%)	0	
**Postoperative rectal injury**			x
-** No**	824 (100%)	160 (100%)	
-** Yes**	0	0	
Postoperative hospitalization (days)	3.75 ± 0.74; 3 (3–7)	3.11 ± 0.36; 3 (3–5)	0.037
Catheterization time (days)	11.53 ± 1.45; 12 (8–14)	9.60 ± 2.06; 10 (6–16)	<0.001
**(b)**			
**Parameter**	**LRP**	**RARP**	***p* Value**
Postoperative Pelvic floor rehabilitation			0.106
- No	230 (81.0%)	125 (78.2%)	
- Yes	54 (19.0%)	33 (21.8%)	
Postoperative PAD test (1 month) (grams)	163.84 ± 222.0; 50 (0–400)	68.17 ± 374.17; 11 (0–404)	0.68
Postoperative PAD test (3 months (grams)	75.57 ± 122.20; 20 (0–480)	14.02 ± 42.09; 0 (0–250)	<0.001
Postoperative PAD test (6 months) (grams)	39.47 ± 76.08; 5 (0–420)	13.62 ± 28.60; 0 (0–80)	0.023
Postoperative PAD test (12 months) (grams)	14.76 ± 29.41; 0 (0–100)	15.33 ± 23.2; 1 (0–50)	0.964
**(c)**			
**Parameter**	**LRP**	**RARP**	***p* Value**
**Nerve sparing technique at surgery**			<0.001
-** No**	223 (78.5%)	94 (58%)	
-** Yes**	61 (21.5%)	66 (42%)	
Monolateral	27 (43.3%)	25 (31.7%)	
Bilateral	34 (56.7%)	41 (68.3%)	0.187
IIEF-5 postoperative (6 months)	9.60 ± 3.78; 9 (6–19)	10.25 ± 3.94; 9.5 (7–15)	0.750
IIEF-5 postoperative (12 months)	10.14 ± 4.77; 10 (5–21)	18.0 ± 3.75; 18 (18–18)	0.117

**Table 3:** Distribution of preoperative and postoperative parameters on the basis of risk classes. Mean ± SD, median, (range). Number of cases (%).

	Low Risk	Intermediate Risk	High Risk	*p* Value
Number cases	142	200	102	
Age (years)	67.73 ± 6.60; 67: (50–73)	67.42 ± 6.61; 68 (49–22)	67.46 ± 6.12; 69 (47–71)	0.906
BMI	25.73 ± 3.70; 25.3 (18–37)	25.84 ± 3.35; 25.4 (18.5–37)	26.81 ± 3.63; 26 (21–39.4)	0.121
Charlson Index	3.82 ± 1.02; 4 (2–7)	3.89 ± 1.08; 4 (0–7)	3.88 ± 1.06; 4 (1–6)	0.903
Familiarity				0.138
- Yes	0	19 (9.5%)	17 (16.7%)	
- No	142 (100%)	181 (90.5%)	85 (83.3%)	
Digital Rectal Examination				<0.001
- Normal	134 (94.4%)	176 (88.0%)	66 (64.7%)	
- Suspicious	8 (5.6%)	24 (12.0%)	36 (35.3%)	
Preoperative total PSA (ng/mL)	6.73 ± 3.12; 6.4 (3.0–19.0)	8.41 ± 3.91; 7.5 (3.0–23.0)	11.74 ± 9.3; 9.3 (3.0–64.0)	<0.001
PSAD	0.14 ± 0.49; 0.13 (0.08–0.26)	0.23 ± 0.17; 0.19 (0.06–0.54)	0.3 ± 0.3; 0.24 (0.11–0.59)	0.048
Prostate volume (cc)	47.78 ± 15.48; 45 (25–120)	47.4 ± 13.72; 45 (20–87)	51.53 ± 16.77; 52.5 (25–90)	0.403
**mMR PIRADS score**				<0.001
(Data available on 208 cases)				
PIRADS 2	6 (10.7%)	0 (0%)	0 (0%)	
PIRADS 3	14 (25.0%)	26 (26.2%)	4 (7.7%)	
PIRADS 4	32 (57.1%)	58 (58.6%)	20 (38.5%)	
PIRADS 5	4 (7.2%)	15 (15.2%)	28 (53.8%)	
Prostate Tumor size (mm) at mMR	10.15 ± 4.78; 9.5 (4.0–30.0)	11.83 ± 4.66; 10 (5.0–27.0)	18.25 ± 8.72; 16 (7.0–39.0)	<0.001
Preoperative CT and bone scan				<0.001
- No	133 (93.7%)	88 (87.5%)	66 (64.8%)	
- Yes	9 (6.3%)	25 (12.5)	36 (35.2%)	
Preoperative PET CT scan				0.027
- No	142 (100%)	195 (97.5%)	97 (95.1%)	
- Choline	0	4 (2.0%)	2 (2.0%)	
- PSMA	0	1 (0.5%)	3 (2.9%)	
**Surgical technique at radical prostatectomy**				0.850
- Laparoscopic	92 (64.8%)	126 (63.0%)	66 (64.7%)	
- Robotic-assisted	50 (35.2%)	74 (37.0%)	36 (35.3%)	
**Operative time (minutes)**	150.50 ± 27.35; 140 (120–300)	155.0 ± 20.06; 157.5 (90–210)	183.51 ± 50.711; 175 (90–300)	<0.001
**Nerve sparing technique at surgery**				<0.001
-** No**	73 (51.4%)	145 (72.5%)	99 (97.0%)	
-** Yes**	69 (48.6%)	55 (27.5%)	3 (3.0%)	
- Monolateral	19 (27.5%)	31 (56.4%)	2 (66.7%)	
- Bilateral	50 (72.5%)	24 (43.6%)	1 (33.3%)	< 0.001
**Extended lymph node dissection**				<0.001
-** No**	142 (100%)	167 (83.5%)	0 (0%)	
-** Yes**	0 (0%)	33(16.5%)	102(100%)	
**Pathological stage (T)**				<0.001
pT2	110 (77.5%)	108 (53.0%)	28 (27.4%)	
pT3a	28 (19.7%)	80 (39.9%)	42 (41.2%)	
pT3b	4 (2.8%)	12 (7.1%)	32 (31.4%)	
**Pathological stage (N)**				<0.001
N0	-	26 (73.1%)	89 (87.2%)	
N+	-	7 (26.9%)	13 (12.8%)	
**ISUP grading at surgery**				<0.001
1	75 (52.8%)	14 (7.0%)	3 (2.9%)	
2	57 (40.2%)	109 (54.5%)	17 (16.7%)	
3	9 (6.3%)	58 (29.0%)	22 (21.6%)	
4	1 (0.7%)	12 (6.0%)	35 (34.5%)	
5	0 (0%)	7 (3.5%)	25 (24.5%)	
**Surgical margin at surgery (R)**				0.003
- Negative	129 (90.8%)	162(80.8%)	76 (74.5%)	
- Positive	13 (9.2%)	38 (19.2%)	26 (25.5%)	
**Positive surgical margin grading**				<0.001
-** 3**	13 (100%)	33 (86.8%)	9 (34.6%)	
-** 4**	0 (0%)	5 (13.2%)	16 (61.5%)	
-** 5**	0 (0%)	0 (0%)	1 (3.9%)	
**Positive surgical margin radial distance (mm)**	2.86 ± 0.945; 3 (2–4)	2.65 ± 0.89; 2.5 (2–6)	3.34 ± 1.25; 3 (1–7)	0.104
Postoperative total PSA (ng/mL) (at 1 month)	0.03 ± 0.08; 0.02 (0.01–1.0)	0.04 ± 0.093; 0.02 (0.01–1.0)	0.12 ± 0.3; 0.03 (0.01–2.0)	<0.001
Biochemical progression				<0.001
- No	136 (95.8%)	183 (91.5%)	72 (70.6%)	
- Yes	6 (4.2%)	17 (8.5%)	30 (29.4%)	
Time to biochemical progression (months)	19.0 ± 15.83; 15 (6–48)	23.88 ± 28.22; 18 (1–120)	15.72 ± 24.12; 6 (1–4120)	0.576
Adjuvant therapy				<0.001
- No	139 (97.9%)	186 (93.0%)	66 (64.7%)	
- Yes	3 (2.1%)	14 (7.0%)	36 (35.3%)	
Adjuvant therapy type				0.290
- RT	3 (100%)	13 (92.9%)	21 (58.3%)	
- RT + ADT	0	1 (7.1%)	15 (41.7%)	

**Table 4:** Distribution of preoperative and postoperative parameters on the basis of pT. Mean ± SD, median, (range). Number of cases (%).

	pT2	pT3a	pT3b	*p* Value
Number cases	246	150	48	
Age (years)	66.76 ± 6.45; 67 (48–71)	68.25 ± 6.587; 68 (47–72)	68.79 ± 6.75; 68 (54–73)	0.032
BMI	25.71 ± 3.56; 25.3 7–37.2)	26.25 ± 3.41; 25.7 (19–39.4)	27.21 ± 3.8; 26.1 (19–37)	0.077
Charlson Index	3.77 ± 1.08; 4 (0–7)	4.05 ± 1.01; 4 (0–7)	3.8 ± 1.05; 4 (1–6)	0.179
Familiarity				0.211
- Yes	14 (5.7%)	15 (10%)	6 (12.5%)	
- No	232 (94.3%)	135 (90%)	42 (87.5%)	
Digital Rectal Examination				<0.001
- Normal	226 (91.9%)	118 (78.7%)	52 (66.7%)	
- Suspicious	20 (8.1%)	32 (21.3%)	16 (33.3%)	
Preoperative total PSA (ng/mL)	7.64 ± 4.52; 6.85 (3.0–48.0)	8.59 ± 4.77; 7.5 (3.0–33.0)	13.55 ± 10.14: 11.2 (4.0–64.0)	<0.001
PSAD	0.19 ± 0.15; 0.15 (0.1–0.50)	0.22 ± 0.15; 0.18 (0.1–0.59)	0.35 ± 0.36; 0.23 (0.1–0.50)	0.112
Prostate volume (cc)	48.0 ± 4.53; 45 (20–120)	49.08 ± 14.4; 47 (24–90)	43.91 ± 15.87; 40 (25–75)	0.56
**mMR PIRADS score**				0.01
PIRADS 2	6 (5.2%)			
PIRADS 3	31 (26.9%)	11 (15.1%)	2 (10.0%)	
PIRADS 4	61 (53.1%)	41 (56.2%)	9 (45.0%)	
PIRADS 5	17 (14.8%)	21 (28.7%)	9 (45.0%)	
Prostate Tumor size (mm) at mMR	10.9 ± 4.78; 10 (4–30)	14.10 ± 5.98; 12 (5–35)	20.53 ± 11.33; 18 (8–39)	<0.001
**Clinical T staging**				<0.001
T1	8 (3.2%)	4 (2.7%)	0	
T2a	29 (11.8%)	1 (0.6%)	0	
T2b	106 (43.1%)	76 (50.7%)	10 (20.8%)	
T2c	96 (39.0%)	51 (34.0%)	26 (54.2%)	
T3a	7 (2.9%)	18 (12.0%)	5 (10.4%)	
T3b	0	0	7 (14.6%)	
**Biopsy outcomes**				<0.01
% positive samples PCa	33.15 ± 21.7.25 (5–100)	48.11 ± 26.58; 44 (2–100)	59.66 ± 29.42; 52.5 (10–100)	
**ISUP grading at biopsy**				<0.001
1	113 (45.9%)	31 (20.7%)	5 (10.4%)	
2	77 (31.4%)	50 (33.3%)	8 (16.7%)	
3	34 (13.8%)	37 (24.7%)	15 (31.3%)	
4	20 (8.1%)	24 (16%)	13 (27.0%)	
5	2 (0.8%)	8 (5.3%)	7 (14.6%)	
**Surgical technique at radical prostatectomy**				0.193
- Laparoscopic	157 (63.8%)	91 (60.7%)	36 (75.0%)	
- Robotic-assisted	89 (36.2%)	59 (39.3%)	12 (25.0%)	
**Operative time (minutes)**	159.02 ± 35.57; 160 (90–300)	160.42 ± 33.21; 160 (90–300)	165.28 ± 26.53; 177.5 (100–200)	0.764
**Pathological stage (N)**				<0.01
N0	11 (100%)	68 (88.9%)	36 (75.0%)	
N+	0 (0%)	8 (11.1%)	12 (25.0%)	
**ISUP grading at surgery**				<0.001
1	85 (34.6%)	7 (4.7%)	0	
2	114 (46.3%)	65 (43.3%)	4 (8.3%)	
3	32 (13.0%)	39 (26.0%)	18 (37.5%)	
4	13 (5.3%)	27 (18.0%)	8 (16.7%)	
5	2 (0.8%)	12 (8.0%)	18 (37.5%)	
**Surgical margin at surgery (R)**				<0.001
- Negative	218 (88.6%)	119 (79.2%)	30 (62.5%)	
- Positive	28 (11.4%)	31 (20.8%)	18 (37.5%)	
**Positive surgical margin grading**				0.001
-** 3**	24 (85.7%)	25 (80.6%)	6 (33.3%)	
-** 4**	4 (14.3%)	5 (16.2%)	12 (66.7%)	
-** 5**	0 (0%)	1 (3.2%)	0 (0%)	
**Positive surgical margin radial distance (mm)**	2.72 ± 0.966; 3 (1–4)	2.67 ± 0.84; 3 (2–5)	3.71 ± 1.28; 3.5 (2–7)	0.01
**PNI at surgery**				<0.001
Positive	103 (41.9%)	123 (82.0%)	43 (89.6%)	
Negative	143 (58.1%)	27 (18.0%)	5 (10.4%)	
**Cribriform/IDC at surgery**				0.001
- Positive	2 (0.8%)	10 (6.7%)	8 (16.7%)	
- Negative	244 (99.2%)	140 (93.3%)	40 (83.3%)	
Postoperative total PSA (ng/mL) (at 1 month)	0.03 ± 0.31; 0.02 (0.01–0.1)	0.05 ± 0.143; 0.02 (0.01–1.0)	0.21 ± 0.419; 0.04 (0.01–2.0)	<0.001
Biochemical progression (number of cases and %)				<0.001
-No	234 (95.1%)	127 (84.7%)	30 (62.5%)	
-Yes	12 (4.9%)	23 (13.3%)	18 (37.5%)	
Time to biochemical progression (months)	34.25 ± 42.26; 15 (3–120)	16.1 ± 11.38; 12 (1–36)	11.28 ± 15.18; 2 (1–48)	0.032
Adjuvant therapy				<0.001
- No	242(98.4%)	133 (88.7%)	16 (33.3%)	
- Yes	4 (1.6%)	17 (11.3%)	32 (62.7%)	
Adjuvant therapy type				0.033
- RT	4 (100%)	14 (82.4%)	19 (59.5%)	
- RT + ADT	0	3 (17.6%)	13 (40.6%)	

**Table 5:** Distribution of preoperative and postoperative parameters on the basis of pN status. Mean ± SD, median, (range). Number of cases (%).

	pN0	pN1	*p* Value
Number cases	115	20	
Age (years)	66.16 ± 6; 67 (47–72)	67.37 ± 6.66; 68 (56–71)	0.40
BMI	26.1 ± 3.3; 25.1 (19–39.4)	27.01 ± 3.86; 26.65 (19–32.8)	0.41
Charlson Index	3.75 ± 1.14; 4 (0–7)	4 ± 0.6; 4 (3–5)	0.454
Familiarity			0.145
- Yes	3 (1.5%)	10 (50.0%)	
- no	201 (98.5%)	10 (50.0%)	
Digital Rectal Examination			<0.001
- Normal	156 (76.5%)	9 (45.0%)	
- Suspicious	48 (23.5%)	11 (55.0%)	
Preoperative total PSA (ng/mL)	8.72 ± 5.29; 7.5 (3.0–48.0)	16.54 ± 8.83; 16 (5.0–30.0)	<0.001
PSAD	0.22 ± 0.175; 16 (0.1–0.6)	0.51 ± 0.441; 0.33 (0.1–0.7)	0.012
Prostate volume (cc)	48.38 ± 15.21; 47 (20–120)	53.11 ± 21.86; 50 (25–90)	0.386
Prostate Tumor size (mm) at mMR	11.94 ± 4.77; 10 (5–28)	27.7 ± 10.47; 30 (11–39)	<0.001
**Clinical T staging**			<0.001
T1	4 (3.5%)	0	
T2a	12 (10.4%)	0	
T2b	45 (39.1%)	3 (15.0%)	
T2c	29 (25.2%)	8 (40.0%)	
T3a	21 (18.3%)	6 (30.0%)	
T3b	4 (3.5%)	3 (15.0%)	
**Clinical N staging**			<0.001
N0	114 (99.1%)	17 (85.0%)	
N1	1 (0.9%)	3 (15.5%)	
Number of suspected lymph node at imaging	2 ± 1; 2 (1–3)	4 ± 1; 4 (3–4)	0.21
Nomograms results (% risk for N+)			
Briganti 2012	24.5 ± 17.12; 17: (2–82)	26.4 ± 15.46; 20: (7–85)	0.175
Briganti 2019	23.4 ± 16.45; 16: (2–82)	26.9 ± 21.36; 21: (4–78)	0.143
**Biopsy outcomes**			0.001
% positive samples PCa	38.78 ± 24.76; 30 (2–100)	72.54 ± 24.7; 75 (35–100)	
**ISUP grading at biopsy**			0.304
1	7 (6.1%)	1 (5.0%)	
2	10 (8.7%)	5 (25.0%)	
3	42 (36.5%)	6 (30.0%)	
4	43 (37.4%)	6 (30.0%)	
5	13 (11.3%)	2 (10.0%)	
**Surgical technique at radical prostatectomy**			0.62
- Laparoscopic	64 (55.6%)	12 (60.0%)	
- Robotic-assisted	51 (44.4%)	8 (40.0%)	
**Operative time (minutes)**	166.01 ± 34.74; 160 (90–300)	170 ± 26.06; 175 (135–220)	0.68
**Pathological stage (T)**			<0.001
pT2	11 (9.6%)	0	
pT3a	68 (59.1%)	8 (40.0%)	
pT3b	36 (31.3%)	12 (60.0%)	
**Number Lymph nodes removed at surgery**			0.60
- Total cases	17.07 ± 6.24; 18 (2–35)	17.95 ± 9.38; 18.5 (6–47)	
**Site of positive lymphnodes**			--
- Obturator	-	20 (100%)	
- External iliac	-	8 (40.0%)	
- Internal iliac	-	9 (45.0%)	
**ISUP grading at surgery**			0.001
1	1 (0.9%)	0	
2	14 (12.2%)	2 (10.0%)	
3	44 (38.2%)	8 (40.0%)	
4	36 (38.3%)	2 (10.0%)	
5	20 (17.4%)	8 (40.0%)	
**Surgical margin at surgery (R)**			0.001
- Negative	83 (72.1%)	11 (55.0%)	
- Positive	32 (27.9%)	9 (45.0%)	
**Positive surgical margin grading**			0.384
-** 3**	19 (59.4%)	4 (44.4%)	
-** 4**	12 (37.5%)	5 (55.6%)	
-** 5**	1 (3.1%)	0 (0%)	
**Positive surgical margin radial distance (mm)**	3.03 ± 1; 3 (1–7)	3.79 ± 1.72; 3 (1–7)	0.136
PNI at surgery			0.02
Positive	70 (60.9%)	4 (15.8%)	
Negative	45 (39.1%)	16 (84.2%)	
**Cribriform**			<0.001
- Negative	105 (91.3%)	14 (73.7%)	
- Positive	10 (8.7%)	6 (26.3%)	
Postoperative total PSA (ng/mL)(at 1 month)	0.05 ± 0.19; 0.02 (0.01–2.0)	0.28 ± 0.376; 0.09 (0.01–1.0)	<0.001
Biochemical progression			<0.001
-No	94 (81.7%)	9 (45.0%)	
-Yes	21 (18.3%)	11 (55.0%)	
Time to biochemical progression (months)	28.3 ± 34.6; 12 (1–120)	4.18 ± 7.37; 3 (1–24)	0.030
Adjuvant therapy			<0.001
- No	85 (73.9%)	6 (30.0%)	
- Yes	30 (26.1%)	14 (70.0%)	
Adjuvant therapy type			<0.001
- RT	24 (80.0%)	4 (28.6%)	
- RT + ADT	6 (20.0%)	10 (71.4%)	

**Table 6:** Distribution of preoperative and postoperative parameters on the basis of surgical margins (SM). Mean ± SD, median, (range). Number of cases (%).

	Negative SM	Positive SM	*p* Value
Number cases	367	77	
Age (years)	67.44 ± 6.62; 68 (47–73)	67.78 ± 6.14; 69 (52–72)	0.68
BMI	25.88 ± 3.44; 25,4 (18.0–39.4)	26.89 ± 4.04; 26.2 (19–37)	0.83
Charlson Index	3.81 ± 1; 4 (0–7)	4.14 ± 0.93; 4 (1–6)	0.7
Familiarity			0.96
- Yes	23 (6.3%)	12 (15.6%)	
- No	344 (93.7%)	65 (84.4%)	
Digital Rectal Examination			0.01
- Normal	315 (85.8%)	61 (79.2%)	
- Suspicious	52 (14.2%)	16 (20.8%)	
Preoperative total PSA (ng/mL)	8.0 ± 1.07; 4 (3.0–7.0)	11.54 ± 8.8; 9.6 (3.0–64.0)	<0.001
PSAD	0.21 ± 0.14; 0.17 (0.1–0.50)	0.28 ± 0.318; 0.18 (0.2–0.59)	0.253
Prostate volume (cc)	47.78 ± 14.65; 445 (20–120)	49.97 ± 15.77; 46.5 (25–90)	0.452
Prostate Tumor size (mm) at mMR	11.98 ± 5.66; 10 (4–39)	16.52 ± 8.67; 14 (5–38)	<0.001
**Clinical T staging**			<0.001
T1	12 (3.2%)	0	
T2a	30 (8.2%)	0	
T2b	160 (43.6%)	32 (42.1%)	
T2c	142 (38.7%)	31 (39.5%)	
T3a	22 (6.0%)	8 (10.5%)	
T3b	1 (0.3%)	6 (7.9%)	
**Clinical N staging**			0.002
N0	366 (99.7%)	74 (96.1%)	
N1	1 (0.3%)	3 (3.9%)	
**Biopsy outcomes**			<0.001
% positive samples PCa	38.31 ± 25.167; 30 (5–100)	54.664 ± 26.78; 50 (2–100)	
**ISUP grading at biopsy**			0.14
1	131 (35.7%)	18 (23.4%)	
2	107 (29.1%)	28 (36.4%)	
3	69 (18.7%)	17 (22.0%)	
4	50 (13.6%)	7 (9.1%)	
5	10 (2.7%)	7 (9.1%)	
**Surgical technique at radical prostatectomy**			0.345
- Laparoscopic	239 (65.1%)	45 (59.2%)	
- Robotic-assisted	128 (34.9%)	32 (40.8%)	
**Operative time (minutes)**	160.03 ± 36.02; 160 (90–300)	160.63 ± 22.39; 160 (120–220)	0.92
**Nerve sparing technique at surgery**		5	0.450
-** No**	259 (70.4%)	8 (74.7%)	
-** Yes**	108 (29.6%)	19 (25.3%)	
-** Monolateral**	42 (38.9%)	10 (52.6%)	
-** Bilateral**	66 (61.1%)	9 (47.4%)	0.230
**Pathological stage (T)**			<0.001
pT2	218 (59.4%)	28 (36.4%)	
pT3a	119 (32.4%)	31 (40.2%)	
pT3b	30 (8.2%)	18 (23.4%)	
**ISUP grading at surgery**			0.006
1	84 (23%)	8 (10.4%)	
2	152 (41.3%)	31 (40.3%)	
3	70 (18.9%)	19 (24.7%)	
4	41 (11.2%)	7 (9.1%)	
5	20 (5.7%)	12 (15.6%)	
**Positive surgical margin site**			--
-** Apex**		31(40.2%)	
-** Lateral**		23 (29.9%)	
-** Basal**		6 (7.8%)	
-** Posterior**		12 (15.6%)	
-** Multiple**		5 (6.5%)	
**Positive surgical margin grading**			--
-** 3**		55 (71.4%)	
-** 4**		21 (27.3)	
-** 5**		1 (1.3%)	
**Positive surgical margin radial distance (mm)**	x	2.97 ± 1.1; 3 (1–7)	--
**PNI at surgery**			0.05
Positive	215 (58.5%)	54 (70.1%)	
Negative	152 (41.5%)	23 (29.9%)	
**Cribriform/IDC at surgery**			0.20
- Positive	12 (3.3%)	8 (10.4%)	
- Negative	355 (96.7%)	69 (89.6%)	
Postoperative total PSA (ng/mL)(at 1 month)	0.04 ± 0.97; 0.02 (0.01–1.0)	0.13 ± 3.4; 0.03 (0.01–2.0)	<0.001
Biochemical progression			<0.001
-No	338 (92.1%)	53 (68.8%)	
-Yes	29 (7.9%)	24 (31.2%)	
Time to biochemical progression (months)	25.45 ± 29.4; 18 (1–120)	9.73 ± 11.57; 4.5 (1–48)	0.022
Adjuvant therapy			<0.001
- No	359 (91.6%)	34 (44.1%)	
- Yes	10 (8.4%)	43 (55.9%)	
Adjuvant therapy type			0.037
- RT	4 (40.0%)	33 (76.7%)	
- RT + ADT	6 (60.0%)	10 (23.3%)	

**Table 7:** Distribution of preoperative and postoperative parameters on the basis of biochemical recurrence (BCR). Mean ± SD, median, (range). Number of cases (%).

	No BCR	Yes BCR	*p* Value
Number cases	391	53	
Age (years)	67.13 ± 6.588; 68 (47–72)	68.62 ± 6.7; 70 (49–73)	0.131
BMI	25.74 ± 3.43; 25 (18–37)	27.21 ± 3.61; 26.7 (21.4–39.4)	0.017
Charlson Index	3.85 ± 1.02; 4 (0–7)	3.86 ± 1.39; 4 (0–7)	0.96
Familiarity			0.12
- Yes	28 (7.2%)	7 (13.2%)	
- No	363 (92.8%)	46 (86.8%)	
Digital Rectal Examination			<0.001
- Normal	340 (86.9%)	36 (67.9%)	
- Suspicious	51 (13.1%)	17 (32.1%)	
Preoperative total PSA (ng/mL)	8.14 ± 5.04; 7.1 (3.0–64.0)	11.37 ± 7.03; 9.65 (4.0–30.0)	<0.001
PSAD	0.2 ± 0.145; 0.17 (0.1–0.59)	0.26 ± 0.48; 0.28 (0.1–0.47)	0.007
Prostate volume (cc)	48.15 ± 14.6; 45 (20–120)	48.55 ± 18.37; 50 (25–90)	0.933
Prostate Tumor size (mm) at mMR	11.95 ± 5.14; 10 (4–30)	23.21 ± 11.18; 20 (10–39)	<0.001
**Clinical T staging**			<0.001
T1	12 (3.1%)	0	
T2a	30 (7.7%)	0	
T2b	173 (44.2%)	19 (35.8%)	
T2c	148 (37.8%)	25 (47.2%)	
T3a	26 (6.7%)	4 (7.5%)	
T3b	2 (0.5%)	5 (9.4%)	
**Clinical N staging**			<0.001
N0	390 (99.7%)	50 (94.3%)	
N1	1 (0.3%)	3 (5.7%)	
**Biopsy outcomes**			<0.001
% positive samples PCa	38.68 ± 25.2; 30 (2–100)	59.21 ± 27.57; 50 (14–100)	
**ISUP grading at biopsy**			<0.001
1	143 (36.6%)	6 (11.3%)	
2	123 (31.4%)	12 (22.6%)	
3	70 (17.9%)	16 (30.2%)	
4	46 (11.8%)	11 (20.8%)	
5	9 (2.3%)	8 (15.1%)	
**Surgical technique at radical prostatectomy**			0.034
- Laparoscopic	243 (62.1%)	41 (77.4%)	
- Robotic-assisted	148 (37.9%)	12 (22.6%)	
**Nerve sparing technique at surgery**			0.002
-** No**	270 (69.1%)	47 (88.7%)	
-** Yes**	121 (30.9%)	6 (11.3%)	
-** Monolateral**	48 (28.9%)	4 (80.0%)	
-** Bilateral**	73 (71.1%)	2 (20.0%)	0.016
**Pathological stage (T)**			<0.001
pT2	234 (59.8%)	12 (22.6%)	
pT3a	127 (32.5%)	23 (43.4%)	
pT3b	30 (7.7%)	18 (34.0%)	
pT4	0	0	
**Pathological stage (N)**			0.014
N0	94 (91.3%)	21 (65.6%)	
N+	9 (8.7%)	11 (34.4%)	
**ISUP grading at surgery**			*p* < 0.001
1	88 (22.5%)	4 (7.5%)	
2	172 (43.0%)	11 (20.8%)	
3	75 (19.0%)	14 (24.4%)	
4	40(11.4%)	8 (15.1%)	
5	16 (4.1%)	16 (20.2%)	
Surgical margin at surgery (R)			<0.001
- Negative	338 (86.4%)	29 (54.7%)	
- positive	53 (13.6%)	24 (45.3%)	
**Positive surgical margin grading**			<0.001
-** 3**	45 (84.9%)	10 (41.7%)	
-** 4**	7 (13.2%)	14 (58.3%)	
-** 5**	1 (1.9%)	0	
**Positive surgical margin radial distance (mm)**	2.67 ± 0.82; 3 (1–4)	3.75 ± 1.35; 3.5 (2–7)	0.001
**PNI at surgery**			<0.001
Positive	225 (57.5%)	44 (83.0%)	
Negative	166 (42.5%)	9 (17.0%)	
**Cribriform/IDC at surgery**			0.217
- Positive	15 (3.8%)	5 (9.4%)	
- Negative	376 (91.2%)	48 (90.6%)	
Postoperative total PSA (ng/mL) (at 1 month)	0.03 ± 0.03; 0.02 (0.01–0.1)	0.24 ± 0.433; 0.07 (0.01–2.0)	<0.001
Time to biochemical progression (months)		18.67 ± 24.57; 12 (1–120)	--
Adjuvant therapy			<0.001
- No	359 (91.8%)	32 (60.4%)	
- Yes	32 (8.2%)	21 (39.6%)	
Adjuvant therapy type			0.042
- RT	26 (81.2%)	11 (55.0%)	
- RT + ADT	6 (18.8%)	10 (45.0%)	

**Table 8:** (**a**) Risk for **extracapsular extention** (pT3a and pT3b) at surgery on the basis of different preoperative parameters at univariate and multivariate analysis. (**b**) Risk for **upgrading** (ISUP 1–2 in ISUP 3–5) at surgery on the basis of different preoperative parameters at univariate and multivariate analysis. (**c**) Risk for **lymp hnode involvement** (pN1) at surgery on the basis of different preoperative parameters at univariate and multivariate analysis. (**d**) Risk for **positive surgical margin** at surgery on the basis of different preoperative parameters at univariate and multivariate analysis. (**e**) Risk for **biochemical progression** after surgery on the basis of different preoperative parameters at univariate and multivariate analysis.

**(a)**						
	**Univariate**			**Multivariate**		
**Parameter**	**OR**	**95%CI**	***p* Value**	**OR**	**95%CI**	***p* Value**
**Preoperative PSA**						
<10 ng/mL	1.0			1.0		
≥10 ng/mL	3.46	2.19–5.44	<0.001	1.79	0.78–4.0	0.16
**PIRADS score**						
3	1.0			1.0		
4	1.98	0.92–4.37	0.079	1.30	0.53–3.15	0.50
5	4.84	1.97–11.93	<0.001	1.70	0.55–5.16	0.30
**Prostate biopsy** +						
monolateral	1.0			1.0		
bilateral	1.40	0.95–2.07	0.087	2.0	1.48–6.22	0.064
**Max percentage PCa tissue per core**				Not included		
<25%	1.0					
25–50%	1.33	0.76–2.35	0.285			
51–75%	1.33	0.76–2.35	0.285			
>75%	2.0	0.75–5.33	0.102			
**ISUP grading**						
1 e 2	1.0			1.0		
3	5.41	3.23–0.97	<0.001	2.10	0.95–5.02	0.06
4–5	10.63	5.79–19.52	<0.001	6.40	1.72–24.44	0.006
**Risk Classes**						
Low	1.0					
Intermediate	1.20	0.87–2.45	0.276	1.0		
High	2.90	1.74–4.89	<0.001	2.80	1.1–6.2	0.04
**(b)**						
	**Univariate**			**Multivariate**		
**Parameter**	**OR**	**95%CI**	***p* Value**	**OR**	**95%CI**	***p* Value**
**Preoperative PSA**						
<10 ng/mL	1.0			1.0		
≥10 ng/mL	3.42	1.65–7.10	<0.001	2.20	0.7–8.69	0.15
**PIRADS score**				Not included		
3	1.0					
4	1.57	0.4–6.09	0.51			
5	0.58	0.56–5.96	0.64			
**Prostate biopsy +**						
monolateral	1.0			1.0		
bilateral	2.06	1.01–4.18	0.042	1.71	0.8–3.54	0.1
**Max percentage PCa tissue per core**				Not included		
<25%	1.0					
25–50%	1.0					
51–75%	1.20	0.66–2.15	0.385			
>75%	1.33	0.75–2.53	0.102			
**Risk Classes**						
Low	1.0					
Intermediate	1.0			1.0		
High	2.90	1.74–4.89	<0.001	3.28	1.45–7.4	0.04
**(c)**						
	**Univariate**			**Multivariate**		
**Parameter**	**OR**	**95%CI**	***p* Value**	**OR**	**95%CI**	***p* Value**
**Preoperative PSA**						
<10 ng/mL	1.0			1.0		
≥10 ng/mL	4.27	1.65–11.02	0.001	1.31	0.63–2.71	0.47
**PIRADS score**						
3	1.0			1.0		
4	1.07	1–1.15	0.10	2.55	1.08–6	0.03
5	1.28	1.08–1.53	0.003	3.97	1.36–11.54	0.01
**Prostate biopsy +**						
Monolateral	1.0			1.0		
Bilateral	5.20	1.48–18.36	0.005	1.35	0.96–1.89	0.78
**Max percentage PCa tissue per core**				Not included		
<25%	1.0					
25–50%	1.0					
51–75%	1.0					
>75%	1.50	0.67–3.39	0.27			
**Risk classes**						
Low	1.0					
Intermediate	1.0			1.0		
High	7.32	2.06–25.94	<0.001	4.50	1.7.11.9	0.002
**Number of lymph nodes removed at surgery**						
<10	1.0			1.0		
10–15	3.36	0.7–12	0.10	1.70	0.62–11	0.5
>15	5.69	1.78–18.17	0.001	4.10	1.009–26.5	0.049
**Nomogram risk**						
≤7%	1.0			1.0		
>7%	1.30	1.07–1.49	0.10	1.61	0.17–15.1	0.60
**Surgical technique**						
Laparoscopy	1.0			1.0		
Robotic-assisted	1.20	0.5–3.15	0.60	2.0	0.34–12.66	0.60
**ISUP grading**						
1 e 2	1.0			1.0		
3	9.45	1.93–46.32	0.003	2.21	3.0–11.52	0.005
4–5	9.28	1.96–43.86	<0.001	5.89	3.0–11.52	<0.003
**pTstage**						
pT2	1.0			1.0		
pT3a	1.08	1.01–1.15	0.012	1.20	0.76–1.96	0.40
pT3b	1.70	1.28–2.25	<0.001	3.34	1.5–7.4	0.003
**(d)**						
	**Univariate**			**Multivariate**		
**Parameter**	**OR**	**95%CI**	***p* Value**	**OR**	**95%CI**	***p* Value**
**Preoperative PSA**						
<10 ng/mL	1.0			1.0		
≥10 ng/mL	3.57	2–5.63	<0.001	2.98	1–8.8	0.04
**Prostate volume**				Not included		
<50 cc	1.0					
≥50 cc	1.04	0.47–2.33	0.90			
**PIRADS score**						
3	1.0			1.0		
4	1.57	0.54–4.54	0.40	0.89	0.2–3.2	0.80
5	3.32	1.08–10.23	0.03	1.90	0.7–9.5	0.20
**Prostate biopsy +**				Not included		
Monolateral	1.0					
Bilateral	1.14	0.68–1.09	0.60			
**Risk classes**						
Low	1.0					
Intermediate	1.0			1.0		
High	1.44	0.81–2.54	0.20	4.98	1.0–22.8	0.40
**Surgical technique**						
Laparoscopic	1.0			1.0		
Robotic-assisted	1.27	0.7–2.1	0.35	1.20	0.5–1.8	0.40
**Nerve sparing tecnique at surgery**						
No	1.0			1.0		
Yes	0.80	0.46–1.44	0.48	2.10	0.5–7.9	0.30
**Operative time**				Not included		
≤120 min	1.0					
>120 min	1.57	0.34–7.27	0.56			
**pTstage**						
pT2	1.0			1.0		
pT3a	2.0	1.15–3.52	0.01	2.0	1.1–3.65	0.20
pT3b	4.60	2.27–9.32	<0.001	4.60	2–10.67	<0.001
**ISUP grading at surgery**						
1–2	1.0			1.0		
3	1.65	0.90–3.0	0.10	1.37	0.73–2.66	0.30
4–5	1.84	1.0–3.41	0.05	1.40	0.74–2.70	0.30
**(e)**						
	**Univariate**			**Multivariate**		
**Parameter**	**OR**	**95%CI**	***p* Value**	**OR**	**95%CI**	***p* Value**
**Preoperative PSA**						
<10 ng/mL	1.0			1.0		
≥10 ng/mL	3.0	1.68–5.57	<0.001	4.12	1.2–19.21	0.007
**PIRADS score**						
3	1.0			1.0		
4	1.05	1–1.09	0.06	1.02	0.9–1.20	0.90
5	1.42	1.17–1.71	<0.001	1.90	0.7–9.50	0.20
**Prostate biopsy +**						
Monolateral	1.0			1.0		
Bilateral	2.64	1.37–5.10	0.003	4.52	0.81–25.10	0.08
**Risk classes**						
Low	1.0					
Intermediate	1.0			1.0		
High	4.35	2.47–8.44	<0.001	9.66	1.85–50.32	0.007
**Surgical technique**						
Laparoscopic	1.0			1.0		
Robotic-assisted	0.38	0.19–0.75	0.007	0.66	0.17–2.55	0.66
**Nerve sparing tecnique at surgery**						
No	1.0			1.0		
Yes	0.24	0.09–0.62	0.002	0.30	0.10–2.20	0.30
**Operative time**						
≤120 min	1.0			1.0		
>120 min	1.10	0.99–1.14	0.23	0.98	0.65–1.62	0.90
**pTstage**						
pT2	1.0			1.0		
pT3a	3.42	1.64–7.14	<0.001	1.20	0.70–3.0	0.40
pT3b	11.10	4.80–25.75	<0.001	2.82	0.99–8.21	0.06
**ISUP grading at surgery**						
1–2	1.0			1.0		
3	3.21	1.47–7.0	0.005	1.87	0.83–5.72	0.190
4–5	6.76	3.32–13.70	<0.001	3.73	1.50–9.50	0.006
**Lymphnode involvement**						
pN0	1.0			1.0		
pN1	2.46	1.82–5.13	0.010	8.32	1.53–45.0	0.014
**Number of Lymph nodes removed**						
<10	1.0			1.0		
10–15	3.18	0.99–10.21	0.60	1.7	0.40–3.20	0.40
>15	2.86	1.24–6.60	0.010	2.2	0.80–4.50	0.20
**Surgical margins**						
Negative	1.0			1.0		
Positive	4.97	2.39–10.34	<0.001	7.20	1.80–28.30	0.004
**Surgical margins grade**						
3	1.0			1.0		
4	5.0	5.57–45.0	<0.001	4.96	2.37–10.37	<0.001
**PNI at surgery**						
Negative	1.0			1.0		
Positive	4.50	1.92–10.55	<0.001	1.50	0.63–4.50	0.32

## Data Availability

The data presented in this study are available on request from the corresponding author.

## Supplementary Materials

The following supporting information can be downloaded at: https://www.mdpi.com/article/10.3390/cancers16213604/s1, Table S1: Characteristics of the whole population included in the study.
