# Supplementary material for: Real-Life Comparative Analysis of Robotic-Assisted Versus Laparoscopic Radical Prostatectomy in a Single Centre Experience
Source: Cancers (Basel). 2024 Oct 25;16(21):3604. doi: 10.3390/cancers16213604 (PMC11545600; doi:10.3390/cancers16213604)
Supplement: Supplementary file 1 [file cancers-16-03604-s001.zip › cancers-3227923-supplementary.pdf]

Supplementary Table S1. Characteristics of the whole population included in the study. Mean±SD, median, (range). Number of cases (%).

Number cases	444
Age (years)	67.49±6.53; 68: (47-73)
BMI	26.05±3.55; 26.0: (18.0- 39.40)
Charlson Index	3.86±1.06; 4: (0-7)
Familiarity<br>- Yes<br>- no	 35 (7.9%)<br>409(92.1%)
Digital Rectal Examination<br>- Normal<br>- Suspicious	 376 (84.7%)<br>68 (15.3%)
Preoperative total PSA (ng/ml)	8.61±5.74; 7.30: (3.0-64.0)
PSAD	0.22±0.186; 0.17: (0.1-0.59)
Prostate volume (cc)	48.17±14.79; 45.0: (20.0-120.0)
**mMR PIRADS score**<br><br>PIRADS 2<br>PIRADS 3<br>PIRADS 4<br>PIRADS 5	(data available in 208 cases)<br><br>6 (2.9%)<br>44 (21.1%)<br>111 (53.4%)<br>47 (22.6%)
Prostate Tumor size (mm) at mMR	12.8±6.53; 11.0: (4.0-39.0)
Preoperative CT and bone scan<br>- No<br>- yes	 374 (84.3%)<br>70 (15.7%)
Preoperative PET CT scan<br>- no<br>- choline<br>- PSMA	 434 (97.7%)<br>6 (1.3%)<br>4 (1.0%)
**Clinical T staging**<br>T1<br>T2a<br>T2b<br>T2c<br>T3a<br>T3b<br>T4	 12 (2.7%)<br>30 (6.7%)<br>192 (43.2%)<br>173 (39.0%)<br>30 (6.8%)<br>7 (1.6%)<br>0
**Clinical N staging**<br>N0<br>N1	 440 (99.1%)<br>4 (0.9%)
Number of suspected lymph node at imaging	3.0±1,4; 3: (2-4)
Prostate biopsy type<br>- Random<br>- Target<br>- Target + random	 163(36.7%)<br>17 (3.8%)<br>264 (59.5%)
Prostate biopsy number of cores	12.61±3.65;12 (4-27)
**Biopsy outcomes**<br>% positive samples PC<br>% positive clinical significant PC<br>Max% PC tissue per core	 41.31±26.13; 34: (2-100)<br>53.47±28.5; 50.0: (8.0-100.0)<br>58.35±24.7; 50.0: (6.0-100.0)

Prostate biopsy laterality +<br>- Monoliteral<br>- bilateral	 203 (45.7%)<br>241 (54.3%)
**ISUP grading at biopsy**<br>1<br>2<br>3<br>4<br>5	 149 (33.6%)<br>135 (30.4%)<br>86 (19.4%)<br>57 (12.8%)<br>17 (3.8%)
**Risk Class (D'Amico**)<br>- Low risk<br>- Intermediate risk<br>- High risk	 142 (32.0%)<br>200 (45.0%)<br>102 (23.0%)
**Nomograms results (% estimated risk for N+) in intermediate and high risk cases**<br>Briganti 2012<br>Briganti 2019	 <br>23.40±19.11; 17.5: (2-85)<br>17.58±31.4; 12.85: (2-95)
**Surgical technique at radical prostatectomy**<br>- Laparoscopic<br>- Robotic assisted	 284 (64%)<br>160 (36%)
**Operative time (minutes)**	160.05±33.73; 160: (90-300)
**Nerve sparing technique at surgery**<br>- **No**<br>- **Yes**<br>- Monolateral<br>- Bilateral	 317 (71.4%)<br>127 (28.6%)<br>52 (40.9%)<br>75 (59.1%)
**Extended lymph node dissection**<br>- **no**<br>- **yes**	 309 (69.6%)<br>135 (30.4%)
**Pathological stage (T)**<br>pT2<br>pT3a<br>pT3b<br>pT4	 246 (55.4%)<br>150 (33.8%)<br>48 (10.8%)<br>0
**Pathological stage (N)**<br>N0<br>N+	 115 (85.2%)<br>20 (14.8%)
**Number Lymph nodes removed at surgery**<br>- Total cases<br>- N+ cases<br>- N0 cases	 17.22±6.81; 18: (4-47)<br>17.95±9.38; 19: (6-47)<br>17.07±6.24; 18: (4-35)
Percentage positive lymph nodes in pN+ cases	14.80 ±11.60; 10.4: (4.0-26.6)
**Site of positive lymphnodes (number of cases)**<br>- Obturator right<br>- Obturator left<br>- External iliac right<br>- External iliac left	 10 (50.0%)<br>13 (65.0%)<br>4 (20.0%)<br>6 (30.0%)

- Internal iliac right<br>- Internal iliac left	7 (35.0%)<br>9 (45.0%)
**ISUP grading at surgery**<br>1<br>2<br>3<br>4<br>5	 92 (20.7%)<br>183 (41.2%)<br>89 (20.1%)<br>48 (10.8%)<br>32 (7.2%)
**Surgical margin at surgery (R)**<br>- Negative<br>- positive	 367 (82.7%)<br>77 (17.3%)
**Positive surgical margin site**<br>- **apex**<br>- **lateral**<br>- **basal**<br>- **posterior**<br>- **multiple**	 31 (40.2%)<br>23 (29.9%)<br>6 (7.8%)<br>12 (15.6%)<br>5 (6.5%)
**Positive surgical margin grading**<br>- **3**<br>- **4**<br>- **5**	 55 (71.4%)<br>21 (27.3%)<br>1 (1.3%)
**Positive surgical margin radial distance (mm)**	2.97±1.1; 3 (1-7)
**Positive surgical margin**<br>- **Single**<br>- **multiple**	 72 (93.5%)<br>5 (6.5%)
**PNI at surgery**<br>positive<br>negative	 269 (60.5%)<br>175 (39.5%)
**Cribriform/IDC at surgery**<br>- positive<br>- negative	 20 (4.5%)<br>424 (95.5%)
**Postoperative hospitalization (days)**	3.41±0.56;3 (3-7)
Catheterization time (days)	10.27±2.0; 10: (6-16)
Postoperative anastomotic leakage (at 30 days)<br>- no<br>- yes	 441 (99.3%)<br>3 (0.7%)
Postoperative blood transfusion (at 30 days)<br>- no<br>- yes	 442 (99.5%)<br>2 (0.5%)
Postoperative lymphocele. (at 30 days)<br>- no<br>- yes	 436 (98.2%)<br>8 (1.8%)
Postoperative anastomotic stricture. (at 90 days)<br>- no<br>- yes	 432 (97.3%)<br>12 (2.7%)
Postoperative rectal injury (at 30 days)<br>- no<br>- yes	 444 (100%)<br>0 (0%)
Postoperative total PSA (ng/ml)(at 1 month)	0.09±0.17; 0.02: (0.01-2.0)
Postoperative follow-up (months)	56.4±37.8; 48 (12-120)
Biochemical progression	

No<br>Yes	391 (88.1%)<br>53 (11.9%)
Time to biochemical progression (months)	18.67±24.57;12 (1-48)
Adjuvant therapy<br>- no<br>- RT<br>- RT+ADT	 391 (88.1%)<br>37 (8.3%)<br>16 (3.6%)
IIEF-5 preoperative (patients submitted to nerve sparing)	20.75±5.83; 23: (12-25)
Postoperative use of tadalafil<br>- No<br>- yes	 317 (71.4%)<br>127 (28.6%)
IIEF-5 postoperative (6 months) (patients submitted to nerve sparing)	9.68±3.74; 9: (6-19)
IIEF-5 postoperative (12 months) (patients submitted to nerve sparing)	10.41±24.10; 8: (5-21)
Penile prothesis<br>- no<br>- yes	 420 (94.6%)<br>24 (5.4%)
Postoperative Pelvic floor rehabilitation<br>- no<br>- yes	 355 (79.9%)<br>89 (20.1%)
**Postoperative PAD test**<br>1 months (grams)<br>3 months (grams)<br>6 months (grams)<br>12 months (grams)	 99.37±33.0; 20.0: (0-404)<br>45.14±95.9; 4.0 (0-480)<br>34.86±70.5; 2.0 (0-420)<br>14.82±28.7; 0: (0-100)
No PADS postoperative status	407 (91.6%)
Artificial Sphincter<br>- no<br>- yes	 432 (97.3%)<br>12 (2.7%)
